# A mixed methods approach for the identification and assessment of workforce innovations in home health care

**DOI:** 10.3389/frhs.2026.1749947

**Published:** 2026-07-16

**Authors:** Luca Grieco, Ken Eason, William Maton-Howarth, Martin Utley, Sonya Crowe

**Affiliations:** 1 Clinical Operational Research Unit, Department of Mathematics, University College London, London, United Kingdom; 2 The Bayswater Institute, London, United Kingdom

**Keywords:** decision support tool (DST), home health care (HHC), operational research (OR), optimisation, sociotechnical systems (STS) theory, soft systems methodology (SSM), workforce

## Abstract

**Background:**

The effective and efficient deployment of staff in home-based health care is fundamental to the sustainability of health and social care systems, particularly in the context of ageing populations and an increasing number of patients with complex needs.

**Methods:**

We combined Soft Systems Methodology, mathematical optimisation and sociotechnical systems analysis of a local health economy in London, UK, to identify and assess the potential impact of different (combinations of) workforce innovations on multiple aspects of system performance, and the readiness of the local health economy to implement them.

**Results:**

Through stakeholder engagement, we identified 21 aspects of system performance and 38 potential innovations that could be considered to improve one or more of them. We quantitatively compared the potential impact on several aspects of system performance of a subset of the innovations chosen because they are seemingly in tension, promote similar goals but at different planning levels, or illustrate intrinsic trade-offs between different aspects of system performance. We additionally assessed the innovations according to the local health economy's readiness for them and the likely barriers to their successful implementation.

**Conclusion:**

Our multifaceted approach synthesises an organisation's preferences and change capabilities, while providing some additional quantitative insights on the potential effects of different innovations under consideration together with a rich understanding of the aspects of system performance considered important by different stakeholders. Decision makers could use this novel approach to explore and assess different combinations of innovations for their organisation.

## 1 Introduction

Avoiding preventable hospital admissions and reducing and delaying care home admissions are widely considered crucial to the sustainability of health and social care systems in the United Kingdom (UK) (1, 2), particularly in the context of an ageing demographic profile. The effective and efficient deployment of staff to deliver home-based care and the greater use of citizen and community assets beyond statutory health and social care organisations are recognised as critical to these aims (3). However, the existing model for deploying staff in home-based health care is stretched and would arguably struggle to cater with projected increases in the number of older patients with complex care needs (4, 5).

The difficulty of meeting rising demand for home health care is exacerbated by constraints on funding and on the availability of workforce, with, for example, the number of district nurses falling by approximately 40% between 2009 and 2019 in the UK (6). In a recent survey of community provider leaders, 76% reported workforce recruitment and retention as the biggest challenge for their organisation, citing exhaustion and low morale following the pandemic, the cost-of-living crisis and rising fuel prices (which impacts on community staff who travel to visit patients) as exacerbating factors (7). This brings a focus to seeking improvements in how the available workforce is deployed.

In a 2013 survey (8), community nurses reported that in their previous 7.5 hour shift they saw 6-10 patients and spent around 85 minutes travelling and 145 minutes on administrative tasks. Eight in ten were dissatisfied with the division of their time and many were concerned about the quality of care due to insufficient contact time resulting in task-focussed rather than holistic care. Seven in ten reported unpredictable and excessive caseloads and workload, which they attributed to poor staffing levels, skill-mix and high sickness rates. In a more recent survey of community nurses (9), 84% reported that working conditions had worsened in the last year, 78% had worked overtime on their last shift and 89.6% of all teams reported that they did not have a full complement of permanent staff on their last shift. An NHS confederation 2022 report (7) stated that the creation of new roles, or alternative routes into existing roles, could go some way to addressing staff shortages and developing a resilient and skilled workforce for the future.

The logistical problems intrinsic to home care mean that it has long been seen as amenable to approaches from operational research [see Utley et al. 2022 (10) for an overview of operational research approaches in health care improvement]. Reviews highlight that the majority of OR studies in home care focus on staff-patient allocation, visit scheduling and staff-routing with the objective of reducing staff travel times (11, 12). Grieco et al. 2021 (11) observed that this technical literature often takes the “who does what” of home care as immutable and so ignores the scope for improvements in the operation of services through the design of new or augmented staff roles. Another finding was that individual studies tended to focus on decisions made at a single level, for instance operational-level decisions on which member of staff ought to conduct which visit, or tactical decisions on team composition, and so ignore the scope for alignment or misalignment of decision making at different levels to support or subvert improvement initiatives.

High-level professional perspectives on how best to meet the challenges of delivering home care are naturally informed by far greater knowledge of the context, structure and delivery of services and of the scope for changing the “who does what”. For instance, the Queen's Nursing Institute reported a feeling among teams that too much complex work was being delegated, and also expresses concern about the trend towards the use of “timed-task” approaches within “apps” or “electronic schedulers” to determine the working days of nurses, making a distinction between this and the use of such analysis to inform or plan workload (13).

Largely missing from both the technical literature and the professional literature on workforce innovations is the extent to which the healthcare communities in question would be ready and able to adopt innovations. There is a considerable literature on readiness for organisational change, which largely focuses on two issues. First, it considers the readiness of employees to adapt to change [for example, Holt et al. 2007 (14)]. Second, it is concerned with strategies to prepare organisations for change [for example, Armenakis et al. 1993 (15)]. Whilst both of these issues are important and relevant to workforce innovation in home health care, they do not cover many other organisational features important in assessing readiness for change. There may be, for example, a requirement for structural changes to the organisations, revisions to work processes or changes to the technical systems that support the work processes (16–18). In parallel, implementation science has developed frameworks to explain how innovations are adopted and sustained in complex healthcare systems. Weiner's theory of organisational readiness for change conceptualises readiness as a shared state of commitment and collective efficacy (16), while broader frameworks such as CFIR (19) and PARIHS (20) emphasise the interaction between intervention characteristics, organisational context and implementation processes. Normalisation Process Theory (NPT) (21) further highlights the work required by individuals and teams to embed new practices into routine care.

The design, evaluation and adoption of innovations in any complex system such as home health care are beset by the fact that different aspects of system performance are valued, prioritised and promoted differently by different stakeholders. Commissioners, patients, provider organisations, clinical and non-clinical staff may all attach different importance to measures such as access times, continuity of care and workload balance, and may bring different notions to the concepts of efficiency and productivity (22, 23).

Operational Research comprises, in addition to quantitative approaches for identifying and evaluating solutions to well-defined problems, methods for understanding multiple perspectives on problems and for understanding those necessary attributes of solutions that are not easily captured within mathematical models. In previous work related to congenital heart disease, we have discussed the combination of quantitative and qualitative Operational Research approaches (24) and in work related to Emergency Department crowding we combined quantitative Operational Research with the use of organisational ethnography to identify solutions likely to be impactful in theory and to be implementable in practice for an organisation (25). Our contribution in this paper is to build on and extend this methodological approach and in doing so address gaps in the literature on workforce innovation. Specifically, we aimed to develop and apply a mixed methods approach combining soft-systems methodology (26), mathematical optimisation and sociotechnical systems analysis to identify potential workforce innovations in home health care organisations, quantify the potential impact of selected innovations on system performance, and assess the organisation's readiness for change with respect to the innovations selected.

We applied this approach to an organisation providing home health care in a borough in London, UK. In particular, soft-systems methodology allowed us to understand different stakeholder perspectives on the performance of home-care services, quantitative optimisation helped us gauge the potential impact of different innovations on different aspects of system performance, including explicit consideration of alignment or misalignment of decision making at different levels, and sociotechnical systems analysis provided insights into the facilitators and barriers to implementation of these potential innovations.

## 2 Materials and methods

In this section we set out the methods we used: to conduct a soft systems analysis to characterise the problem, understand multiple perspectives on aspects of system performance and to identify potential solutions intended to improve one or more aspect of efficiency; to apply a suite of quantitative Operational Research decision models for estimating the scale of impact that a health economy might obtain if applying a specific innovation or set of innovations; and to conduct a sociotechnical systems analysis for understanding the readiness for change and likely barriers to successful implementation of selected innovations. The work was carried out within the context of a local health economy in London, UK, comprising two Boroughs served by a National Health Service (NHS) community and mental health care provider.

### 2.1 Soft systems methodology

Over a 26-month period we had discussions with a range stakeholders, attended workshops, undertook non-participatory observation of meetings and observed staff as they allocated patient visits to the available staff. Two of the research team (SC and MU) and several of the wider study team had existing working relationships with individuals and organisations within the local health economy studied and initial discussions were with individuals drawn from these networks, who made recommendations of other stakeholders we should talk to.

Standard approaches from soft system methodology (27) such as the development of CATWOES, root definitions and rich pictures (26) were used to surface and make explicit the different perspectives on home health care system performance held within different groups and organisational entities. These interim research artefacts were then used along with meeting notes and documentary analysis to:

- describe the distinct aspects of system performance that emerged from this process, classify them where possible according to accepted definitions of efficiency (22) and dimensions of quality (28), and identify attendant metrics
- construct a set of descriptions of candidate workforce innovations

We then mapped the candidate innovations to the aspects of system performance they are intended to promote.

### 2.2 Selection of innovations to study further

To demonstrate the approach of using the quantitative modelling framework (Sections 2.3, 2.4) alongside the sociotechnical systems analysis (Section 2.5), we focused for detailed study on a set of innovations from those identified through the soft systems analysis. In particular, we first selected the innovations that could be analysed using the current version of the quantitative modelling framework. We then refined the selection by defining groups of comparative analyses involving innovations that seemed to conflict (in terms of their intended goal or mechanism) or that share an intended goal but operate at different planning levels. We limited this selection to innovations for which we had access to quantitative data for model parameterisation.

### 2.3 Assessing innovations using the quantitative modelling framework

**Figure 1:**
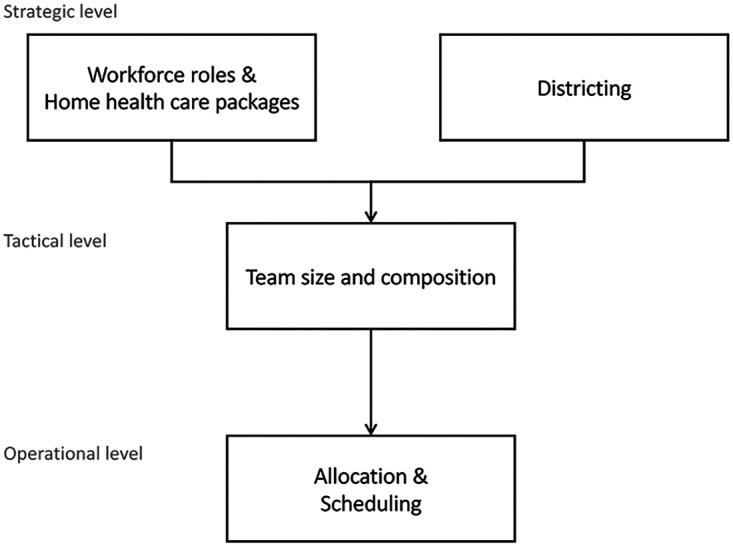
Diagrammatic representation of the hierarchy of decisions considered in our analyses.

Our quantitative assessment of the selected innovations was based on the modelling approach described in Grieco et al. 2025 (29), who developed a hierarchically organised suite of quantitative models designed to address the following strategic, tactical and operational decision problems concerning the delivery of home health care (Figure 1):

- ‘Workforce roles & Home health care packages’ (strategic) - Which combinations of activities to group into different types of visits and which activities may be allocated to which workforce roles (by specialty and grade) to deploy during the planning period, based on a goal such as minimising the total number of visits required to satisfy the expected medium-term or long-term demand.
- ‘Districting’ (strategic) – Which wards in a territory to group together into a pre-defined number of ‘districts’ that define the geographical areas over which different care teams operate, based on a goal such as minimising travel distances for staff.
- ‘Team size and composition’ (tactical) – How many staff members in each role (specialty and grade) need to be available on any day in the planning period, for a given district, based on a goal such as minimising staff costs.
- ‘Rostering, Allocation & Scheduling’ (operational) – Which set of patient visits each available member of staff will conduct for a given week in the planning period and district, and the day of the week that each visit has to be conducted, based on a goal such as minimising staff costs.

The modelling tool builds decision problem instances by extracting parameter values from a dataset whose structure closely matches the type of data usually available as routinely collected home health care patient records. Inputs to and outputs from each decision are also defined along with the precedence relationships between decisions so as to enable a consistent way of exploring the potential benefits from applying mathematical programming or heuristic approaches (10) to individual decisions or to combinations of decisions. Importantly, the specification of each decision problem includes an expression of the goal of the decision maker with respect to that decision.

For each of the selected innovations in this study, we specified the mathematical programming formulations or heuristic algorithms to be used in the framework, the hierarchical order in which to tackle the decision problems, the set of performance metrics to assess, and a dataset representing the innovation scenario of interest.

Analyses were run by executing a set of scripts in the open-source programming language R that read relevant information from the dataset, create the problem instance, solve the problem and give as output the solution and estimated performance across several system metrics. Supplementary File 1 provides a description of the mathematical programming and heuristic approaches we used in our analyses.

### 2.4 Synthetic data

While the modelling tool developed by Grieco et al. 2025 (29) can be instantiated by directly using real patient record data, it also comprises a synthetic data generator that enables construction of simulated patient record data with desired characteristics. We decided to run our analyses using synthetic data generated using this tool for two reasons. Mainly, this approach removed all challenges related to data governance while still using realistic data. Additionally, the exercise of systematically defining features for generating simulated data that are representative of a given health economy in a given scenario makes exploration and comparison of alternative settings much easier and supports reproducible analyses spanning different planning levels.

The synthetic dataset, which is generated using inputs specified for the local health economy of interest, comprises a set of demand features (a list of synthetic patients with their geographical location and the type and frequency of home care activities each patient requires for each week in a reference planning period) and a set of service features (a list of activities provided by the home health care organisation and their service times; a list of workforce roles with salary rates and the set of activities staff in each role may perform; groupings of activities into “visit types”, with corresponding visit times).

We generated the synthetic data starting from patient records from two boroughs in London over the period 01/01/2018 to 31/12/2018. In Supplementary File 1, we describe the procedures we used to parameterise the synthetic data generator and to verify consistency of the simulated data with the real ones. We produced one separate dataset instance for each analysis, with scenarios differing in terms of home health care services delivered and/or workforce features (see Section 3.2).

The quantitative results reported in this paper are therefore rooted in the geographical, demographic and workforce features of our health economy of interest. These include the distribution of home health care demand across a territory of interest, working time regulations, activity/visit durations, workforce structure, travel characteristics (e.g., urban vs rural areas). Such features were all parameterised using available data and information from our collaborating home health care providers, and can be adapted to represent health systems with different characteristics by amending the parameters of the synthetic data generator and/or of the quantitative decision models.

### 2.5 Sociotechnical systems analysis of organisational readiness to change

To assess the readiness for change in the community healthcare services in the local health economy studied, we conducted an overarching sociotechnical systems analysis of the current organisation of services. This focused on major structural elements such as how the workforce was divided into different specialist teams and how they were distributed across each borough, and on their ‘openness’ in terms of the nature of any cooperation with social services, domiciliary care and others. We then conducted an analysis of recent change programmes to assess the nature and scale of the changes involved and the capacity of the systems to make these changes. For each programme, we identified factors that were facilitators or barriers/inhibitors and assessed the extent to which changes became embedded within normal delivery of services.

Based on this analysis, we then developed a checklist for assessing organisational readiness to change using a distinction from sociotechnical systems theory (30) to differentiate between factors that apply at an organisational level (termed “distal”) and factors that apply in the immediate setting of candidate innovations (termed “proximal”). We applied this checklist to the set of chosen innovations, specifically in the context of one borough. To summarise the findings of this process, we classified the innovations in terms of ease of initial launch and, separately, in terms of ease of embedding change.

### 2.6 Presentation of findings to stakeholders

As a final step of the soft systems methodology, we presented our findings on the potential benefits and likely acceptability of distinct innovations to a group of relevant frontline staff and managers and discussed how our findings could be relevant to their current plans and initiatives for improving services.

## 3 Results

### 3.1 Aspects of systems performance and candidate innovations in home care

A summary of our data gathering for the soft systems analysis is shown in Table 1. As examples of the interim artefacts produced through the soft systems analysis, we include the Rich Picture in Supplementary File 2 and the CATWOES and root definitions for the perspectives of healthcare provider organisation, health care professionals, patients and healthcare commissioning organisation in relation to district nursing services in Supplementary File 3.

**Table 1:** Summary of data gathering for the soft systems analysis.

Activity	Group size	Stakeholder group(s)													Total
		Service managers (health)	Service managers (social care)	Service managers (health and social care)	Health care professionals	Social care professionals	Commissioning organisations (social care)	Commissioning organisations (health care)	Domiciliary care managers	Community-based assets	Informal carers	Patients	Health and social care managers and professionals	Health care professionals and managers	
Informal interviews/discussions	1	6	0	8	2	0	7	0	4	2	1	1	0	0	**31**
	2	1	0	0	1	2	1	3	0	0	0	0	0	1	**9**
	3	0	0	0	0	0	1	0	0	0	0	0	0	1	**2**
	>3	1	0	0	0	0	0	0	0	0	0	0	0	0	**1**
**Sub-total**		**8**	**0**	**8**	**3**	**2**	**9**	**3**	**4**	**2**	**1**	**1**	**0**	**2**	**43** *
Observations of work	1	0	0	0	2	0	0	0	0	0	0	0	0	0	**2**
Workshop (non-participant observation)	≈10	1	0	0	0	0	0	0	0	0	0	0	2	0	**3**
Workshop (active participation)	≈10	1	0	0	0	0	0	0	2	0	0	1	0	0	**4**
**Total**		**10**	**0**	**8**	**5**	**2**	**9**	**3**	**6**	**2**	**1**	**2**	**2**	**2**	**52**

* among 53 unique participants.

Based on these discussions, meetings and observations and our soft systems analysis, the aspects of home care system performance we identified are shown in Table 2. Supplementary File 4 further reports potential metrics for these aspects of performance, whether they relate to allocative, productive or technical efficiency and/or which of the Institute of Medicine's domains of quality (28) they relate to, the sphere(s) of influence they come under and the stakeholder groups that raised them. The 21 aspects of performance provide, collectively, a comprehensive and nuanced view of how the goals and limitations of services (individually and in combination) are perceived by overlapping groups of stakeholders. Both the tensions between stakeholder perspectives and the intrinsic relationships and trade-offs between different system metrics caution against an overly reductive view of system performance and its improvement.

**Table 2:** Aspects of system performance identified based on discussions, meetings, observations and soft systems analysis.

Aspects of system performance
How the time of clinical staff is spent
How the competencies of staff are deployed
Staff skills mix
Times between referral, assessment, and access to services
Cognisance and use of other support available to individual patients
Outcomes and costs outside of home care sector
Longer-term impacts
Patients scheduled to be seen at times convenient to them
Punctuality of home visits
Cancellations and postponements
Continuity of relationships
Continuity of information
Integration and coordination of care
Patient contact time
The safety and efficacy of services/treatments
The timing and accuracy of reassessments
The number of patients treated within budget and to an acceptable standard
The sustainability of the workforce
Patient experience
Equity of provision
Degree of personalisation

The candidate innovations that emerged through the soft systems work are listed in Table 3, with Supplementary File 5 providing their descriptions as well as the stakeholder groups that promoted them. We identified 38 distinct changes to services that could be considered as innovations or interventions to improve one or more aspects of system performance. These ranged from the (seemingly) straightforward idea to inform patients by text message when a professional due to visit them was running late, through greater use of technology and the introduction of new or altered roles to the workforce, to changing the entire basis on which services are commissioned.

**Table 3:** List of candidate innovations.

Candidate innovations		
Grouping wards into more compact districts to reduce anticipated travel times for staff	Team size and composition chosen to meet demand at lowest cost	Joint commissioning of health and social care from a pooled budget
Grouping wards into districts in a way that balances anticipated workload across districts	Team size and composition chosen to promote workload balance across staff	Use of advanced computational algorithms to support rostering
Place-based care - grouping wards into districts that have similar sized populations and borders aligned with political entities and primary care catchment areas	Use of patient acuity scores to promote workload balance within teams	Increased use of personal health budgets
Reducing repetition of tasks by several staff working with the same patient	Each patient having a named key worker as first point of contact with oversight of care from multiple teams	Systematic use of social prescribing and signposting
Development of joint health and social care plans for patients	Coordination of home-visits across different teams within health service and across health services and domiciliary care	Personalisation of social care plans
Making explicit and extending the roles of informal carers	Goal-driven allocation of staff to visits, scheduling of visits and routing of staff between visits	Outcomes-based commissioning
Enhanced care workers - training domiciliary care workers in simple nursing tasks	Promotion of “agile working” through for example increased staff use of portable devices	Trusted assessors - training domiciliary care workers to assess evolving needs of clients
Enablement champions - training domiciliary care workers in supporting work of therapy teams	Improved quantitative assessment of population needs across services and teams	Increased use of personal care budgets
Greater flexibility in who does what and a shift to more generalist roles	Active, goal-driven queue management	The “three conversations model” of needs assessment, early intervention and drawing on personal strengths and community assets
Widening accreditation for assessing the need for and ordering of equipment for patients’ homes	Increased focus on early intervention and prevention	Virtual clinics and telehealth
Introducing the role of a nursing and therapy support worker	Offering patients choice of visit times	Transporting home-bound patients to clinics
Introducing a nursing role that covers both mental and physical health	Shared access to electronic health and social care records	Introducing non-clinical roles to support reablement and address social isolation
Increased specialisation with staff working predominantly at the upper end of their competencies	Messaging patients when staff running late	

All but one of the 21 aspects of system performance identified through the soft systems analysis were deemed to be a driving motivation for at least one of the candidate innovations, the exception being “patient contact time”. In Supplementary File 6, we map each candidate innovation to one or more motivating aspect of system performance and indicate whether the innovation could be evaluated using the quantitative modelling framework.

A third of the innovations identified through the soft systems analysis were changes that we felt could be evaluated using the modelling framework, namely:

- Innovation 1. Specialisation: Increased specialisation with staff working predominantly at the upper end of their competencies.
- Innovation 2. Generalisation: Greater flexibility in who does what and a shift to more generalist roles.
- Innovation 3. Balancing workload through districting: Grouping wards into districts in a way that balances anticipated workload across districts.
- Innovation 4. Balancing workload through team size and composition: Team size and composition chosen to promote workload balance across staff.
- Innovation 5. Balancing workload through automated allocation with fuzzy boundaries: Goal-driven allocation of staff to visits and visit scheduling with the goal specified as balancing workload among post-holders for each role within a team.
- Innovation 6. Enhancing continuity of care through automated allocation: Goal-driven allocation of staff to visits and visit scheduling with the goal specified as maximising continuity of care.
- Innovation 7. Minimising staff costs through automated allocation: Goal-driven allocation of staff to visits and visit scheduling with the goal specified as minimising staff costs
- Innovation 8. Balancing continuity of care and costs through automated allocation: Goal-driven allocation of staff to visits and visit scheduling with the goal specified as balancing staff costs and continuity of care.

We explored these in three separate analyses: Analysis 1 examined innovations 1 and 2, chosen as innovations seemingly in tension; Analysis 2 examined innovations 3, 4 and 5*,* chosen as innovations promoting similar goals but at different planning levels; Analysis 3 compared innovations 6, 7 and 8, chosen to illustrate intrinsic trade-offs between different aspects of system performance.

### 3.2 Quantitative analysis results

#### 3.2.1 Analysis 1

The decision models related to workforce roles and the composition of home health care packages were used in combination to study the differences offered by specialisation vs generalisation of staff in terms of skill use, the number of patient visits made, staff time deployed on visits and the related costs. Here, specialisation was interpreted as staff spending more of their time working at the upper end of their competencies, and enacted within the modelling framework by staff not being allocated to conduct activities that could alternatively be conducted by a member of staff in a role of a lower grade. See Supplementary File 1 for a description of this combined decision model (“Workforce roles & Home health care packages” formulation).

We focused on demand for District Nursing, Mental Health and Physiotherapy services: the synthetic data generated covered 4,856 patients, with 39,837 requests for District Nursing activities, 2,861 requests for Mental Health activities and 2,034 requests for Physiotherapy activities over a reference planning period of 52 weeks.

The different degrees of specialisation were defined through a “role-to-activity” matrix defining which roles may conduct which activities:

- Innovation 1 (Specialisation): Each activity is conducted by a member of staff in the lowest grade role allowed to perform the activity
- Innovation 2a (Generalisation within specialty) (baseline): Higher grade staff can be allocated activities that could also be conducted by staff of a lower grade, within the same service type
- Innovation 2b (Generalisation across specialties): As in 2a but also including activities belonging to different service types, namely: District Nursing staff above a given grade can conduct selected basic Mental Health or Physiotherapy activities; Mental Health staff above a given grade can conduct selected basic District Nursing activities or Physiotherapy activities; Physiotherapy staff above a given grade can conduct selected basic District Nursing or Mental Health activities.

In the analysis, a visit time is determined by the sum of the estimated times required for each activity forming that visit, plus an “overhead” time accounting for activities not directly related to the performing of health care tasks (e.g., establishing a rapport with the patient to put their care needs in the broader context of their life), which we assumed to be 6 minutes. We also included an average travel time between patient locations, which we estimated as 11 minutes for the London borough studied, based on the synthetic dataset. To explore how the comparison between innovations might differ in a rural rather than urban setting (as in our London borough), we also performed the analysis assuming an average travel time between patient locations three times as long (i.e., 33 minutes) (‘rural’ scenario).

**Figure 2:**
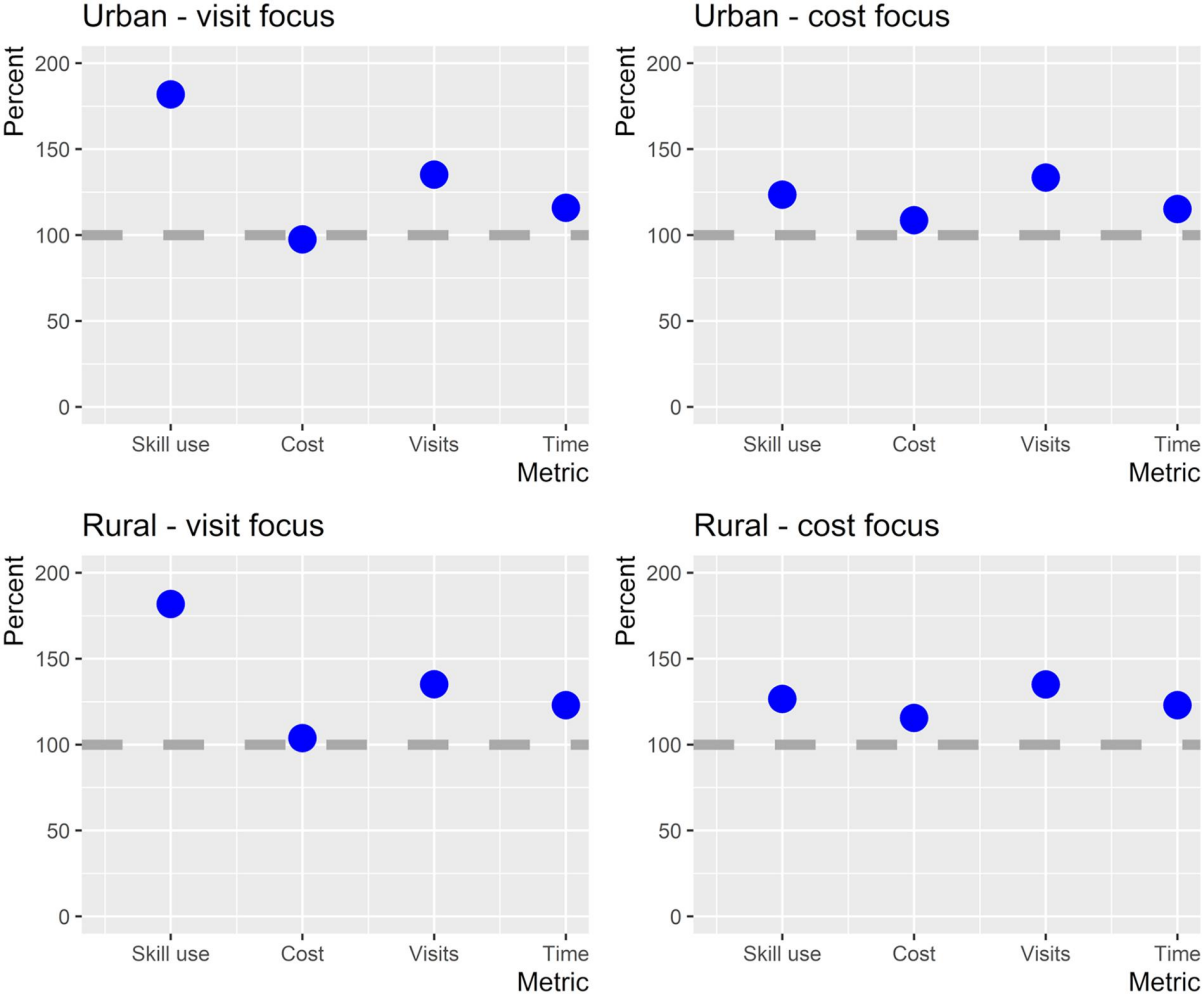
Charts showing comparisons of model output on skill use (proportion of time spent by staff doing activities at their highest skill level), cost (annual visit costs), visits (number of visits required annually) and time (hours spent by staff on visits annually) under specialisation (innovation 1), presented as a percentage of the equivalent values obtained under generalisation within specialty (innovation 2a).

Figure 2 shows a summary of the differences that emerged from this analysis. In each chart, the value obtained under specialisation (Innovation 1) for each of the four metrics considered is presented as a percentage of the equivalent value obtained under generalisation within specialty (Innovation 2a, our baseline).

By definition, specialisation (Innovation 1) gives a higher level of skill-use across all analyses and also results in more visits being made (and more clinician time being used). If generalisation is used to minimise the number of visits made, it gives a marginally higher cost than specialisation in the urban setting and a marginally lower cost in the rural setting. If generalisation is used to minimise cost, generalisation is cheaper than specialisation in both urban and rural settings and the difference in skill-use is less marked.

Note that generalisation that includes staff of one specialty taking on some activities of another specialty (Innovation 2b) gives results that differ only very slightly from generalisation solely within specialty (Innovation 2a). In particular, results for the former are almost identical to the latter (0.03% to 0.84% variation from baseline) in all scenarios depicted in Figure 2, with the exception of a variation of −7.4% in skill use when the number of visits is minimised. See full tabulated results at https://github.com/lucagrieco19/workforce_innovation_assessment.

In short, the benefits or otherwise of the generalisation of roles is crucially dependent on both the context of service delivery and trade-offs between different aspects of system performance.

#### 3.2.2 Analysis 2

Decision models related to the partition of a region into districts, team size and composition, the initial allocation of individual staff to patient-visits and short-term fixes to staff-visit allocations were used to study the impact of different approaches to address notions of balanced workload.

We conducted this analysis on a synthetic dataset covering the demand for District Nursing only, which comprised 4,104 patients and 41,749 requests for District Nursing activities over a reference planning period of 52 weeks.

We determined the workforce roles and the home health care packages by solving our Workforce roles & Home health care packages formulation for the whole borough of interest, with minimisation of the total number of visits to cover the annual demand assuming the role-to-activity mapping as in innovation 2a (generalisation within specialty). We then determined solutions to the sequence of decision problems: Districting → Team size and composition → Allocation & Scheduling. For each step we defined a workload balance metric to be optimised, in order to analyse the effects of innovations with aligned aims but acting at different planning levels. In particular, we set:

- the Districting formulation to maximise the balance of expected annual activity time across the resulting districts;
- the Team size and composition formulation to maximise the balance of worked hours among the number of salaried staff deployed in each role;
- the Allocation & Scheduling formulation to maximise the balance of worked hours among the individual staff members deployed in a specific week, further refined by visit swaps to balance within-role worked hours across districts.

We also defined “no-optimisation” versions of the solution algorithms for each of the three decision problems (see Supplementary File 1), to simulate situations in which the corresponding innovation is not introduced. For Districting, no-optimisation corresponds to the current subdivision of the territory into three districts. For Team size and composition, the no-optimisation case was calculated using a heuristic that determines the number of staff members that should be available daily by allocating the average daily demand (inflated by 10% to account for some variability) for each visit type to a randomly chosen role picked among the cheapest roles that could be assigned that visit type. For Allocation & Scheduling, the no-optimisation solution was determined using a heuristic procedure that randomly assigns visits to available staff (salaried staff while available, and then agency staff) across the week until the demand is satisfied.

We conducted four sets of analyses. In the first analysis [decision path (a)], we solved the Districting problem optimally and then we used the no-optimisation algorithms for Team size and composition and for Allocation & Scheduling. For the second analysis [decision path (b)], we considered a non-optimised Districting, we then optimised Team size and composition, and finally we used the no-optimisation algorithm for Allocation & Scheduling. In the third analysis [decision path (c)], we did not optimise Districting and Team size and composition, but we optimised Allocation & Scheduling. Finally, we ran an analysis [decision path (d)] where the three decisions in the sequence were made without optimisation.

**Figure 3:**
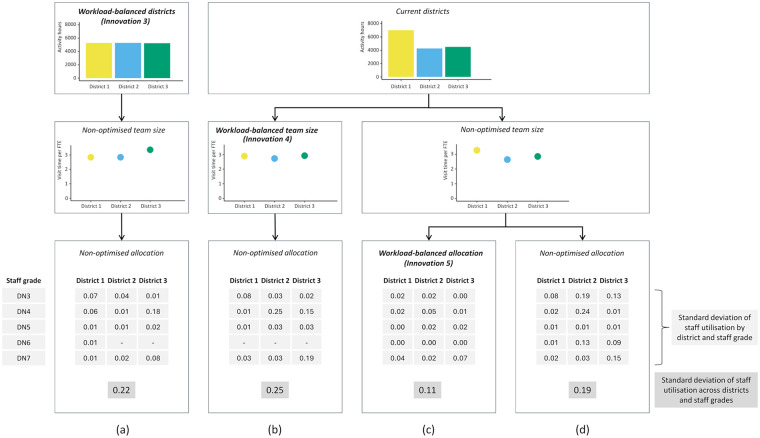
The balance of workload in terms of overall activity-time (top – districting decision), in terms of visit-time per full-time-equivalent (FTE) member of the across districts team (middle – team size and composition decision), and in terms of the standard deviation of staff utilisation (bottom – allocation & scheduling decision) under different combinations of practice [i.e., the four decision paths **(a-d)** indicated by the arrows]. Staff grade labels DN3 to DN7 denote the role “District Nurse” with bands 3 to 7, respectively.

Figure 3 shows a summary of the results obtained. It can be seen that the use of a Districting optimisation algorithm can be effective in balancing the overall workload (in terms of total expected activity time) between districts, but that this balance does not translate into a very balanced workload within and between district level teams in the absence of other initiatives downstream in the chain of decisions [Figure 3 – decision path (a)]. Similarly, innovation in determining team size and composition can deliver workload balance between district-level teams despite the unbalanced activity hours across districts, but with no guarantee that this will translate to workload balance within teams [Figure 3 – decision path (b)]. The use of an optimisation algorithm in allocating staff to visits that permits allocations of staff to patients across district boundaries (“allocation with fuzzy boundaries”) is shown to have potential to balance individual workload across grades and districts despite the workload balance performance metrics upstream in the chain of decisions are not optimised [Figure 3 – decision path (c)].

By exploiting the hierarchical structure of the decision making process, this type of analysis provides insights on the extent to which strategic or tactical decisions affect operational decisions, providing the decision maker with a tool to assess effective alignment or tension between performance metrics used at different planning levels.

#### 3.2.3 Analysis 3

Decision models on the allocation of staff to patient-visits, with different choices of objective, were used to show the potential benefits of algorithm-informed operational decision making and the nature of trade-offs between notions of continuity of relationships and staffing costs.

We conducted this analysis on a synthetic dataset covering the demand for District Nursing and Mental Health services, which comprised 4,888 patients with 41,898 requests for District Nursing activities and 2,999 requests for Mental Health activities over a reference planning period of 52 weeks.

We adopted the ‘no innovation’ formulations in the ‘Districting’ (“no-optimisation” in Analysis 2), ‘Workforce roles & Home health care packages’ (baseline in Analysis 1) and ‘Team size and composition’ (“no-optimisation” in Analysis 2) decisions. We then solved the Allocation & Scheduling problem for a randomly sampled week in the planning period in the following cases (see Supplementary File 1 for details):

- *No optimisation* – a heuristic procedure randomly assigns visits to available staff (first salaried staff, then agency) across the week until demand is satisfied.
- *Enhancing continuity of care (Innovation 6)* – the goal (objective function) of the algorithm is to minimise the number of unique staff-patient pairs across the week.
- *Minimising staff costs (Innovation 7)* – the goal (objective function) of the algorithm is to minimise the staff costs (salaries + agency staff pays) across the week.
- *Balancing continuity of care and staff costs (Innovation 8)* – the goal of the algorithm is to minimise the equally weighted (normalised) sum of the objective functions defined at the two points above.

The trade-off between staff monetary costs and continuity of care as defined for this analysis are shown in Table 4. Of particular note is that the use of goal-driven allocation gave fewer patient-staff pairings (our metric for continuity of care) and lower costs than our “no innovation” case regardless of whether the goal specified was minimum cost, fewest pairings or a compromise between these. Secondly, setting an objective for goal-driven allocation of staff to visits that seeks a balance between costs and continuity of care makes a good improvement in continuity at modest cost increase.

**Table 4:** Unique staff-patient pairings and staff monetary costs for a randomly sampled week in the planning period, associated with each of the four choices of objectives considered in analysis 3.

Case	Number of unique staff-patient pairings	Staff monetary costs
No innovation	1,120	£15,170
Enhancing continuity of care (Innovation 6)	880	£14,960
Minimising staff costs (Innovation 7)	1,060	£14,540
Balancing continuity of care and staff costs (Innovation 8)	900	£14,630

This analysis showcases a tool for exploring the trade-off between conflicting aspects of system performance that act at the same planning level.

### 3.3 Readiness for change and barriers to implementation

In developing the “readiness for change” checklist, we examined 16 recent change programmes across two boroughs, including changes to district nursing team structures, training domiciliary care workers in some “health” tasks, and the introduction of new services, new workforce roles and information technology.

The checklist developed is fully described in Supplementary File 7. It involves:

- the identification of factors likely to enable or inhibit a specific change that operate at the level of a whole system or organisation in general (termed the distal perspective);
- the identification of the local system of work processes, staff groups, relationships and information flows, etc. impacted by a specific change under consideration;
- the identification of factors likely to enable or inhibit the specific change that operate at the level of this local system (termed the proximal perspective).

**Figure 4:**
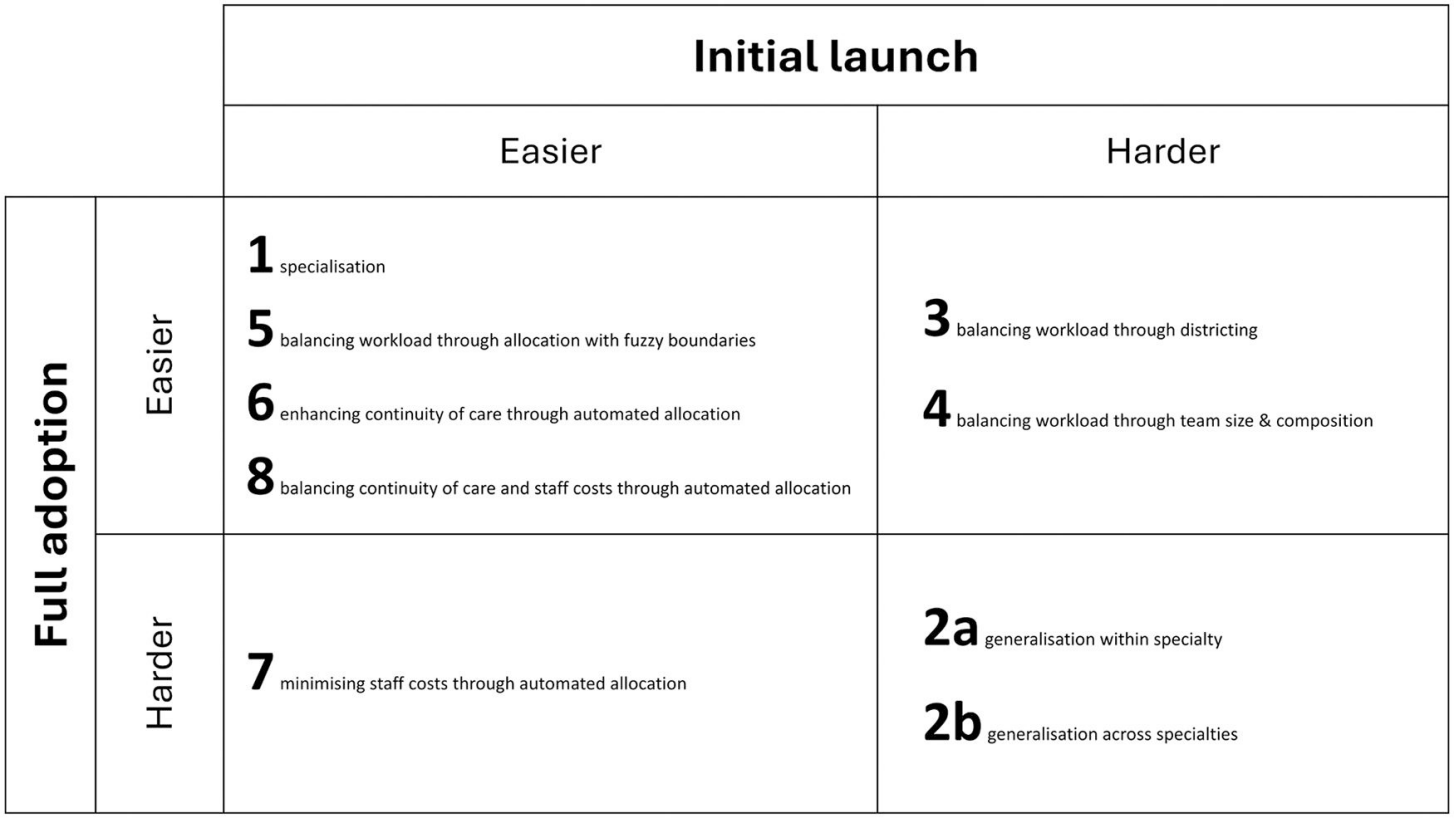
The eight selected innovations compared by ease of initial launch and by ease of full adoption within the case-study health-economy, as determined through the sociotechnical systems analysis.

A summary of the assessment for the eight innovations is provided in Supplementary File 8, with full results available at https://github.com/lucagrieco19/workforce_innovation_assessment. Figure 4 further summarises the results from application of the checklist by comparing the innovations by ease of their initial launch and by ease of full adoption.

Most of the innovations need both technical system development and organisational work before they can be launched. No attempt has been made here to assess which of the technical developments will be the most challenging to accomplish and the position on the initial launch scale is based only on the organisational and workforce changes that are necessary.

The biggest organisational change and the hardest to implement is likely to be re-districting to balance workloads (innovation 3) followed by innovation 4, which may involve district nursing staff moving localities to change the size and composition of the teams. Other innovations do not envisage planned organisational changes but some could potentially involve significant role changes and changes in clinical practice for healthcare staff. Innovations 2a and 2b, for example, enlarge the range of duties of healthcare staff. Innovation 2a is likely to be easier to implement than 2b because the enlargement of duties is restricted to one specialty and does not involve the member of staff performing duties for another specialty. Of the three workforce re-balancing innovations, innovation 5 will be the easiest to implement because no staff transfers between teams are envisaged although arrangements will need to be agreed for some out-of-district visits to be undertaken. The three innovations (6, 7 and 8) that specify priorities for allocating visits should not be difficult to launch within the organisation because they only involve a technical system proposing visit allocations to the existing teams delivering healthcare. The innovations that prioritise continuity of care (6 and 8) may be easier to launch than innovation 7 because minimising costs may have more implications for existing ways of making visit allocations. The easiest innovation to implement should be innovation 1 because it reinforces what is already common practice by matching grades to the tasks to be undertaken in visits.

But launching an innovation is only part of the ‘readiness to change’ story. Following the launch, there is the question of the extent to which the workforce actually adopts the innovation and enables the planned benefits to be achieved. A consideration of these issues changes the place of many of the innovations in the scale in Figure 4. In the majority of the cases the local workforce have discretion over the extent to which they adopt the suggestion of technical systems. The exceptions are the two organisational changes (innovations 3 and 4) in which the adoption process requires only that staff adopt their normal roles albeit in different districts or teams. On the basis of the existing evidence, it seems likely that achieving full adoption will be most difficult in innovation 2b, closely followed by 2a, because the adoption of more generalist roles involves changes to many of the current role and career norms in community healthcare. By contrast, innovation 1 may be the easiest to adopt because the specialisation approach is already accepted practice. Innovation 5 may be relatively quickly adopted if it really helps to resolve fluctuations in caseload provided there are mechanisms by which any concerns of the district teams involved have about specific out-of-district visits can be resolved. Similarly, innovation 6 and 8 may also be relatively quickly adopted if they enable continuity of care to be achieved for visits where the community healthcare staff consider it important. By contrast, innovation 7 may prove difficult to embed as normal practice if minimising costs means that other criteria that the workforce consider important such as continuity of care have to be abandoned.

Although innovations may differ in the degree of difficulty there may be in their adoption, there are ways of managing the difficulties in the adoption process and improving the chances of success in relation to all of them. For example, in the cases where the issue is the degree to which local teams adopt the recommendations of technical systems, a sustained programme of formative evaluation within an action research strategy [for example, Argyris et al. 1985 (31) and Klein and Eason 1991 (32)] could serve to assess reasons for non-acceptance and plan actions accordingly.

### 3.4 Results summary

The quantitative modelling and qualitative sociotechnical systems analysis provide useful insights when taken together. For instance, the finding from the sociotechnical analysis that the candidate innovation of adopting more generalist roles in which staff from one discipline perform some of the simpler tasks currently performed by staff of other disciplines is likely to be very difficult to launch and to embed is compounded by the finding from the quantitative modelling that it would bring very little benefit beyond the candidate innovation where the activities performed within generalist roles are restricted to those currently performed within a member of staff's home discipline.

As another example, while the candidate innovation of redistricting to balance workload across teams has potential to be effective, the finding from the quantitative modelling that this potential may be squandered unless reinforced through team sizing and then operational allocation decisions is compounded by the (intuitive) finding from the sociotechnical analysis that it would be very problematic to achieve in the first instance, and that the candidate innovation of “fuzzy boundaries” to smooth out workload between teams might be easier to launch and be at least as effective.

### 3.5 Feedback to stakeholders

The workshop participants felt that many of the innovations in the analyses above were or had been considered. For example, they were interested in a technical visit allocation and scheduling solution, and had tested one such product but found that it did not sufficiently capture some important patient characteristics and had also encountered information governance issues. A different product was being piloted by the community healthcare provider in a neighbouring borough. One issue flagged as potentially problematic in allocation/scheduling technology was that it might lack the flexibility to incorporate important service ambitions such as scheduling a team lead visit at regular intervals (e.g., every third visit). However, the ability to allocate and schedule visits over a longer time horizon (e.g., 1 week rather than 1 day) was seen as a major advantage of technical solutions as staff find it frustrating that they do not know what their visits are beyond the next day.

Regarding the possibility of staff working across disciplinary boundaries, the participants noted that nurses were increasingly undertaking some therapy activities and working better with mental health colleagues. However, whilst they saw value in working with greater flexibility, they felt that the skills development required would be challenging to achieve whilst staff were so busy. In particular, it was noted that there was a shortage of experienced nursing staff (some had left during the pandemic), leading to challenges in clinical oversight of junior staff and patients concerned that they never see a senior nurse and feel a lack of continuity of care. Regarding specialisation, it was noted by participants that staff get burnt out when they are constantly working at the higher end of their specialty, which could lead to long term sickness and poor staff wellbeing. This could be exacerbated by the current shortage of senior nursing staff. The participants also noted that any innovations would need to be considered alongside existing change programmes being undertaken.

## 4 Discussion

The planning, organisation and delivery of home-based care for the elderly is complex and difficult, with intrinsic logistical challenges running alongside tensions between different system goals and scope for a different emphasis on system goals among and between professional groups and the organisations they work within. This multifaceted piece of research attempted to address this complexity and has produced a set of useful insights.

Our use of soft systems methodology yielded a rich understanding of the very many aspects of system performance that occupy those working within and receiving home care services and the candidate innovations for system improvement that have been or could be considered, and crucially mapped the innovations to the particular aspect(s) of system performance they target. A broad view emerged on what could be considered as aspects of system performance, and we note that some of these went beyond standard classifications of efficiency within economics and, by incorporating staff-side measures, beyond the IoM domains of quality.

Using the modelling approach described in Grieco et al. 2025 (29), we were able to conduct a comprehensive analysis of the innovations considered in our study, estimating impact across a number of key performance metrics to understand any trade-offs. In particular, the hierarchical nature of the quantitative framework enabled analysis of sequential decisions at different planning levels so that we could estimate the effects of strategic decisions on operational decisions, and the use of a common underlying synthetic dataset with desired features allowed us to compare different scenarios in a consistent way. This allowed us to explore how the potential benefits of an innovation vary with the context of the home-care system (the overall scale of the workload or for shorter versus longer average travel times between patients).

We took this approach one step further by exploring the readiness for the specific changes associated with the considered innovations among the organisations we worked with. While the range of innovations considered was limited by their amenability to quantitative modelling, by the current features of the modelling tools and by availability of data from the collaborating home health care organisation, we were able to test the potential of our mixed-method approach and obtain useful insights about the case study analysed. This innovative combination of methods synthesises an organisation's preferences and change capabilities while providing some additional (quantitative) insights on the potential effects of the different innovations under consideration.

As well as completing our assessment of the potential impact of innovations in specific problem contexts in specific organisations, the readiness for change checklist developed through this work has stand-alone value in allowing teams within health and social care with responsibility for implementing change to assess the work that they need to undertake [a full guide is available at (33)].

The quantitative modelling approach provided a comprehensive view of the potential effects of the different innovations. Taking this comprehensive approach introduced, almost inevitably, some limitations in the specificity with which we were able to address constituent decision problems in this work. For instance, in our use of data we chose to represent the needs of patients as falling into one of a limited number of profiles, and care as following standardised pathways, losing some of the richness of individual need and some of the holistic nature of home care.

While some degree of reductionism is part and parcel of any operational research modelling exercise, it is important to remember that the nuance lost may be important to the work modelled and to the professional identity of those doing the work. This does not argue against using operational research approaches, but does argue for using them with care and in collaboration with the professions affected. The concerns raised by Rosenhead half a century ago (34) remain relevant.

Managerialist approaches that reduce a visit to a patient by a registered nurse to the set of constituent tasks performed, and ascribe to each task an average value for how long that task *should* take (“timed-task approaches”) have been severely criticised within the nursing profession as leading to staff discontent and resignation (13).

Several of the candidate innovations discussed in this work are rooted in similar world view and so care is required to ensure that the insights and potential benefits of such approaches serve rather than undermine the professions. For instance, it is possible to account as we did for additional, value-adding activity by staff that is not directly attributable to named tasks. Further, leveraging the stochastic capabilities of the synthetic generator to account for the intrinsic patient-to-patient variability and uncertainty in the time that a visit may take would support the development and evaluation of algorithms and other solution approaches that are better matched to the full complexity of home health care.

Fundamentally, solutions offered by algorithmic approaches have the potential to account for the complexity of home health care systems and can offer solutions that would find best quantitative trade-offs between different aspects of system performance. However, professionals should feel empowered to assess such solutions, and perhaps adapt or even reject them, depending on the specific context of their organisation. Used in that spirit, quantitative modelling of the sort deployed in this work can make a valuable contribution to improvements in home health care.

Our study has policy relevance beyond the UK context. The approach provides a basis for explicit policy-level prioritisation of workforce innovations by linking expected system impacts with an assessment of implementation feasibility. In doing so, it may help identify where policies driven primarily by anticipated efficiency gains are unlikely to deliver if misaligned with organisational capabilities and work practices. This has direct relevance for workforce planning and the development of community healthcare models internationally, where decisions must reconcile system-level ambitions with the practical constraints of delivery that apply in local contexts.

## 5 Conclusion

In this study, we combined qualitative and quantitative techniques to identify potential workforce innovations in home health care, quantify their impact on different aspects of system performance, and understand the readiness for change and likely barriers to successful implementation of such innovations. To the best of our knowledge, this is the first study combining Soft Systems Methodology, mathematical optimisation and sociotechnical systems analysis in an attempt to identify and assess workforce improvements in home health care. We believe that this novel multifaceted approach is promising for wider application areas as it provides decision makers with a comprehensive and contextualised view of their improvement options.

## Data Availability

The datasets presented in this study can be found in online repositories. The names of the repository/repositories and accession number(s) can be found in the article/Supplementary Material.

## Supplementary material

Supplementary File S1 Mathematical programming, heuristic approaches and synthetic data generation for the quantitative analysis.

Supplementary File S2 Rich Picture.

Supplementary File S3 CATWOES and root definitions.

Supplementary File S4 Aspects of system performance, with metrics, type of efficiency, sphere of influence and stakeholder group that raised them.

Supplementary File S5 Candidate innovations that emerged through the soft systems analysis.

Supplementary File S6 Mapping of candidate innovation to motivating aspects of system performance.

Supplementary File S7 “Readiness for change” checklist.

Supplementary File S8 Summary of sociotechnical systems analysis results.
