# Supplementary material for: A mixed methods approach for the identification and assessment of workforce innovations in home health care
Source: Front Health Serv. 2026 Jul 16;6:1749947. doi: 10.3389/frhs.2026.1749947 (PMC13422396; doi:10.3389/frhs.2026.1749947)
Supplement: Supplementary File S1 — Mathematical programming, heuristic approaches and synthetic data generation for the quantitative analysis. [file Datasheet1.pdf]

Mathematical programming, heuristic approaches and synthetic data generation for the quantitative analysis

Contents

1 Mathematical programming and heuristic approaches used in the quantitative analyses of the candidate innovations 2
1.1 Workforce roles & Home health care packages 3
1.1.2 Optimised Workforce roles & Home health care packages 3
1.2 Districting 3
1.2.1 Non-optimised Districting 3
1.2.2 Optimised Districting 3
1.3 Team size and composition 4
1.3.1 Non-optimised Team size and composition 4
1.3.2 Optimised team size and composition 4
1.4 Allocation & Scheduling 5
1.4.1 Non-optimised Allocation & Scheduling 5
1.4.2 Optimised Allocation & Scheduling 5
2 Synthetic data generation 6
2.1 Generating synthetic data 6
2.2 Synthetic data verification 7
2.2.1 Comparing the overall annual demand 8
2.2.2 Comparing frequencies of need-weeks among patients 9
2.2.3 Checking the correlation on combinations of activities 11
References 12

1 Mathematical programming and heuristic approaches used in the quantitative analyses of the candidate innovations

We used combinations of "optimisation" models and "no-optimisation" models across the sequence of decisions of interest to mimic, respectively, adoption or non-adoption of the corresponding innovations.

The table below summarises the models we used for each decision problem considered in our set of analyses (cf. Figure 1 in the main text). The following pages provide a more detailed description of each model and relevant references.

**Decision problem**	**No-optimisation version**	**Optimisation version**
Workforce roles & Home health care packages	N/A	Define visit types as combinations of activities and select workforce roles by minimising either expected overall number of annual visits or expected overall salary costs.
Districting	The territory is divided into 3 districts corresponding to the current structure in our reference borough.	The territory is divided into 3 districts by balancing the expected annual activity time across the resulting districts.
Team size and composition	Daily team size is determined for each district using a heuristic procedure randomly assigning each visit to the cheapest role that could conduct all that visit's activities.	Daily team size is determined by minimising staff costs while ensuring workload balance among salaried staff in each role.
Allocation & Scheduling	For a randomly chosen week of the reference planning period and for each district, patient visits are assigned to staff members and days of the week using a heuristic procedure that randomly assigns visits to available staff.	For a randomly chosen week of the reference planning period and for each district, patient visits are assigned to staff members and days of the week by minimising staff cost, minimising the number of unique staff-patient pairs, or maximising workload balance within each role. The solution is then refined by visit swaps to balance within-role workload across districts.

1.1 Workforce roles & Home health care packages

Decision problem: define visit types as combinations of activities and select workforce roles to deploy during the planning period.

1.1.2 Optimised Workforce roles & Home health care packages

This decision consists of selecting a set of workforce roles to deploy in a district over a given planning period (one year in our case studies), while combining the required activities into visit types. The model, described in Grieco et al. 2025 [1], works at the level of “weekly patient profiles”, i.e. defining the set and composition of visits to be delivered to a given patient based on the set of activities the patient needs in a given week (in general, a single patient can “move” between different weekly profiles week by week). Roles are assigned to visits at the same time as visit definition, based on required worker skills. Depending on the placed assigned to this problem in the hierarchical structure, this problem can be either solved for the whole territory of interest (as in our case studies), or separately for each of the districts defined in the districting decision problem (if the districting decision comes earlier in the sequence of decisions).

The problem is solved by minimising either the total staff costs (based on hourly salaries) or the total number of visits required to satisfy the demand over the planning period.

1.2 Districting

Decision problem: given a set of basic units (i.e. wards) defining the territory (i.e. borough) of interest, group those wards into a pre-defined number of clusters each representing a district.

1.2.1 Non-optimised Districting

The territory is divided into a pre-defined number of districts (3 in our case studies) corresponding to the current districting structure in our reference borough.

1.2.2 Optimised Districting

The territory is divided into a pre-defined number of districts (3 in our case studies) by balancing the expected annual workload required across the resulting districts. We measure the workload in terms of expected total activity time required to satisfy the annual demand.

In the districting model formulation, adapted from Dugošija et al. 2020 [2], we consider the set of basic units to be used as building blocks for a specified number of districts (or clusters), their pairwise distances (centroid to centroid) and whether or not they share borders with each other (pairwise contiguity).

The annual activity demand in a basic unit is given by multiplying the estimated number of times a given activity will be requested in that basic unit over one-year time by the expected duration of an

activity of that type. Note, demand is considered at the level of activities and not at the level of visits (i.e. no “overhead” time considered), and it excludes travel time.

We define the deviation between each district’s total demand (in terms of annual activity time) and the average demand across districts (total demand divided by the envisaged number of districts). We minimise the maximum of those deviations to achieve a balance in annual demand across the districts.

1.3 Team size and composition

Decision problem: for a given district, determine what is the number of staff members in each role that have to be available any day in the planning period.

1.3.1 Non-optimised Team size and composition

Daily team size is determined for each district using a heuristic procedure that randomly assigns each required visit to one of the cheapest roles that could conduct all that visit’s activities. To account for variability in the daily numbers of required visits, we inflated the daily demand by 10%.

1.3.2 Optimised team size and composition

Optimal daily team size is determined using a two-stage approach. We adapted an integer linear programming formulation proposed by Rodriguez et al. 2015 [3]. We solve the problem for each district separately, though our implementation also allows considering more districts at the same time.

The second-stage problem optimises team size and composition in a daily scenario and it is solved for a set of daily scenarios picked randomly (20 daily demand scenarios in our case study). We consider daily demand (in terms of visits) at the level of wards and assigns routes (sequences of wards to visit) to different staff members based on their skills. Each staff member can work a limited amount of time in a day (depending on staff role – note, time dedicated to home visits is in general a fraction of the overall staff working time), including time for visits, time to move between wards and time to move between patient locations in a given ward. The objective function minimises the total staff costs formed of a fixed cost per salaried staff deployed and a variable cost per agency staff deployed (with rates depending on staff band and including travel time), while promoting balance of workload (daily hours worked including visit time and travel time) among salaried staff members in the same role.

The first-stage problem considers the second-stage problem solutions and looks for an overall solution “covering” at least a given percentage of the daily scenarios (95% in our case study). In particular, it minimises the salaried (i.e. team) staff costs while ensuring that enough salaried staff members are deployed for at least a given fraction (95% in our analyses) of the scenarios analysed in the slave problem.

1.4 Allocation & Scheduling

Decision problem: for a given week in the planning period and for a specified district, decide which days each available staff member conducts visits, assign each staff members a set of patient visits and decide on which day of the week each visit has to be conducted.

Note, to determine the staff members available on each day (i.e. rostering decision), we start from the required team size and composition, we generate the corresponding minimum number of staff members required in each role and assuming that each staff member is available for no more than five days a week, and then we randomly assign staff members to days such that the team size and composition is constant throughout the week [1].

1.4.1 Non-optimised Allocation & Scheduling

We use a heuristic procedure that randomly assigns each patient visit to an available staff (salaried staff until available, and then agency staff) and to a day of the week in which that staff member is available, until the weekly demand is satisfied. Once the maximum workload for all staff members in a role is reached, the additional visits are assigned to a generic agency staff in the same role.

1.4.2 Optimised Allocation & Scheduling

The Allocation & Scheduling formulation [1] allocates visits to staff members over the short term (planning period of 7 days in our case study) based on their skills, with a time resolution of 1 day (i.e. each staff member is assigned a set of visits each day, but without an hourly scheduling of visits). The demand is specified over a week (7 days) by the set of visits that each patient in a district should receive during that week. Note, due to computational complexity, in these case studies we relaxed the constraints requiring time lags (minimum and maximum elapsed time) between visits to the same patient.

In our analyses, we considered three possible objective functions, briefly described below.

For Analysis 2:

a) minimising staff costs while ensuring workload balance among salaried staff in each role separately – the solution is then refined using a swapping heuristic that re-allocates visits in border-wards to staff from the bordering district in an attempt to improve workload balance among salaried staff in each role across all districts.

For Analysis 3:

b) minimising the total staff costs formed of a daily fixed cost per salaried staff available and a variable hourly cost per agency staff deployed (with rates depending on staff band and including travel time, the latter approximated by an average travel time between visits in a given ward);

c) maximising continuity of care, which is achieved by minimising the number of unique patient-staff pairs (only for salaried staff) over the 7 days.

2 Synthetic data generation

In this section, we describe the procedures we used to parameterise the synthetic data generator and to verify consistency of the simulated data with the real ones.

2.1 Generating synthetic data

The synthetic data generator described in Grieco et al. 2025 [1] takes as input a list of parameters based on which it creates an instance formed of patient needs, service features and geographic features. We estimated such parameters using patient records from two boroughs in London with similar sociodemographic characteristics that were provided by a collaborating organisation delivering home health care in the area.

Due to the presence of incomplete data records, we used data from both boroughs to estimate the parameters, but then generated data dimensioned to match the size and territory of one borough only.

The first step consisted in merging separate datasets containing information about referrals (456,723 records), contacts (996,038 records) and activities (1,777,001 records) delivered by the home health care provider over a period of about four years. The data were made consistent in terms of field names, merged based on common identifiers. We decided to restrict the following parameter estimation on the 52-week time window going from 01/01/2018 to 30/12/2018. The dataset covered 374,311 records and 11,416 patients. From this dataset, we extracted the expected number of patients across the 52 weeks, that is 5,120 for our borough of interest.

To estimate more specific demand features, we then removed all records characterised by unknown referral reason, manually curated activity names, labelled the records based on service types ("District Nursing", "Mental Health", "Physiotherapy"), and further trimmed the dataset by removing records with unspecified activity names. The final dataset used for parameter estimation was formed of 201,932 records, covering 6,544 patients across the two reference boroughs.

The synthetic data generator requires specification of possible "patient states", consisting of sets of activities each patient might require during a week in the planning period together with the number of times each activity is required. The input to the generator includes an initial distribution of patient states (i.e. how frequent each state is) as well as "state-switch" probabilities that specify the chances of a patient moving from a set of needs to another set of needs week after week. The generator also supports stratification of patients into "types" characterised by different states/frequencies/switch probabilities. Therefore, we grouped patients into types based on the set of referrals they underwent during the time window of interest. We discarded the rarest sets of referrals (i.e. associated with less than ten patients each) and the corresponding patients. We obtained 31 patient types. For each of them, we defined patient states based on the activities they went through during the time window of interest and we computed the frequency of each state across all patients of that type. We used these as estimations of the probability of a patient of that type being in each state at the beginning of the planning period. Then, for each ordered pair of states of each patient type, we determined the frequency of switch between those two states by counting the number of times any patient moved between those two states in two consecutive weeks during the whole planning period.

To define the patient base and locations for the specific borough of interest, we used publicly available data. The file "London-wards-2018.zip", published by the Greater London Authority and available at https://data.london.gov.uk/dataset/statistical-gis-boundary-files-london, contains geographic data at ward level (particularly, geographic boundaries and classification into local authority districts) across London in a format that can be exploited by geographic information system (GIS) software, including packages developed in R programming language. We used this dataset as a basis for generating patients in the different wards of the borough.

To determine the staff roles and the types of activities they can conduct, we extracted the roles and bands of staff deployed as reported in the data records considered, and then determined whether a staff of a given role/band is recorded as carrying out a given activity at least once in the analysed dataset. For each role/band, we then retrieved hourly wages from publicly available National Health System data.

Finally, we estimated service time for each activity by considering the staff-patient contacts in the dataset during which that activity was conducted – we first estimated the expected activity time by dividing the contact time by the number of activities carried out during that contact and then, for each activity, we averaged the times estimated above across the whole dataset.

2.2 Synthetic data verification

Here we report on the checks we conducted to verify that the synthetic data generated using our method are consistent with the reference real data. In particular, we verified the patient demand data as these were characterised by randomness in the synthetic data generation process.

Comparing directly the specific patterns of patient needs over time is particularly challenging for this type of data, therefore we used the following approach:

1. We first assessed whether the overall demand for each activity is consistent between real and synthetic datasets.
2. Then, we computed the distribution of "need-weeks" among patients for each activity, that is how many weeks a year each patient needs that activity.
3. Finally, we checked the correlation between the real and synthetic datasets on the combinations of activities requested by patients.

To conduct this analysis we used:

- The trimmed dataset used for parameter estimation formed of 201,932 records, covering 6,544 patients across the two reference boroughs
- 20 synthetic datasets generated using parameters jointly estimated for the two boroughs

2.2.1 Comparing the overall annual demand

We compared the total number of times each activity is needed over one year, across all patients, in the synthetic data versus the real data.

The table below reports the annual demand for each activity (number of times the activity was delivered) in the real data as well as the percentage deviation of the corresponding demand in the synthetic data with respect to the real data, averaged across the 20 datasets generated.

The overall average percent deviation across all activities, weighted by the extent of real-world demand, is 6.94%.

Activity	Annual demand (real dataset)	Average percent deviation from real annual demand in the 20 synthetic datasets
District Nursing - Advice for district nursing	39568	2%
District Nursing - Medication	36143	12%
District Nursing - Wound care	25262	2%
District Nursing - Ulcer care	22635	2%
District Nursing - Blood monitoring	18845	15%
Therapy - Rehabilitation	6703	7%
District Nursing - Daily living	6532	6%
District Nursing - Support	4645	4%
District Nursing - Bandaging	4459	5%
District Nursing - Assessment	4199	5%
District Nursing - Catheter care	4050	8%
District Nursing - Treatment	3569	2%
District Nursing - Diabetic care	3006	20%
District Nursing - Palliative care	2136	5%
Mental Health - Mental health monitoring	1703	11%
District Nursing - Observations	1558	5%
District Nursing - Equipment assessment	1525	4%
Therapy - Review	1402	9%
District Nursing - Post operative care	1267	11%
Therapy - Advice for physiotherapy	901	7%
Therapy - Physiotherapy assessment	816	8%
District Nursing - Liaison	751	6%
Mental Health - Medication	688	10%
Therapy - Occupational therapy assessment	613	9%
Mental Health - Memory assessment	534	26%
Mental Health - Mental health assessment	534	19%
District Nursing - Bladder care	447	28%
District Nursing - Prevention of admission	429	7%
District Nursing - Health promotion	414	20%
District Nursing - Reassessment	404	5%
Mental Health - Risk management	383	12%
Mental Health - Reassessment	322	12%
Mental Health - Carer support	313	14%
District Nursing - Continence assessment	310	15%
District Nursing - End of life care	271	9%
District Nursing - Bowel care	218	17%
District Nursing - Doppler assessment	217	13%
District Nursing - Feeding management	201	28%
District Nursing - Treatment programme	191	7%

Activity	Annual demand (real dataset)	Average percent deviation from real annual demand in the 20 synthetic datasets
Mental Health - Physical health checks	187	10%
Mental Health - Care coordination	171	11%
District Nursing - Supervision of medication	156	12%
District Nursing - Central line care	155	17%
District Nursing - Risk assessment	125	13%
Mental Health - Independence	123	15%
Therapy - Screening	123	16%
Mental Health - Anxiety management	114	25%
Mental Health - Psychoeducation	96	12%
Mental Health - Build confidence	95	24%
District Nursing - Nutritional advice	93	6%
District Nursing - Discharge planning	83	12%
District Nursing - Carer support	82	10%
Mental Health - Care staff support	78	25%
District Nursing - Continence advice	72	18%
District Nursing - Bereavement support	66	14%
Therapy - Mobility therapy	61	14%
District Nursing - Pulmonary rehabilitation	56	43%
Mental Health - Adaptations	43	21%
Mental Health - Accomodation	41	18%
District Nursing - Patient training	33	26%
Mental Health - Relapse prevention	31	15%
Mental Health - Care programme	31	11%
Mental Health - Social inclusion	29	24%
District Nursing - Hydration	29	18%
District Nursing - Gastrostomy	27	24%
Mental Health - Enhance potentials	26	19%
District Nursing - Accident prevention	23	24%
Mental Health - Falls prevention	21	25%
District Nursing - Mobility assistance	20	44%
District Nursing - Chemotherapy	19	24%
Therapy - Physio discharge planning	16	17%
Mental Health - Nutritional advice	14	27%
District Nursing - Tracheostomy care	14	54%
Therapy - Balance therapy	8	57%
District Nursing - Safeguarding adults	3	42%
District Nursing - Funeral attendance	2	65%
Mental Health - Safeguarding adults	1	55%

2.2.2 Comparing frequencies of need-weeks among patients

In this second step we checked, for each patient, how many weeks over the year they need a given activity. We counted how many patients need that activity for 1 week, how many patients need it for 2 weeks, for 3 weeks, etc. therefore obtaining a distribution of “need-weeks” for that activity among patients over the year.

The following figure shows some examples of comparisons of such distributions in the real data versus randomly chosen synthetic data instances.

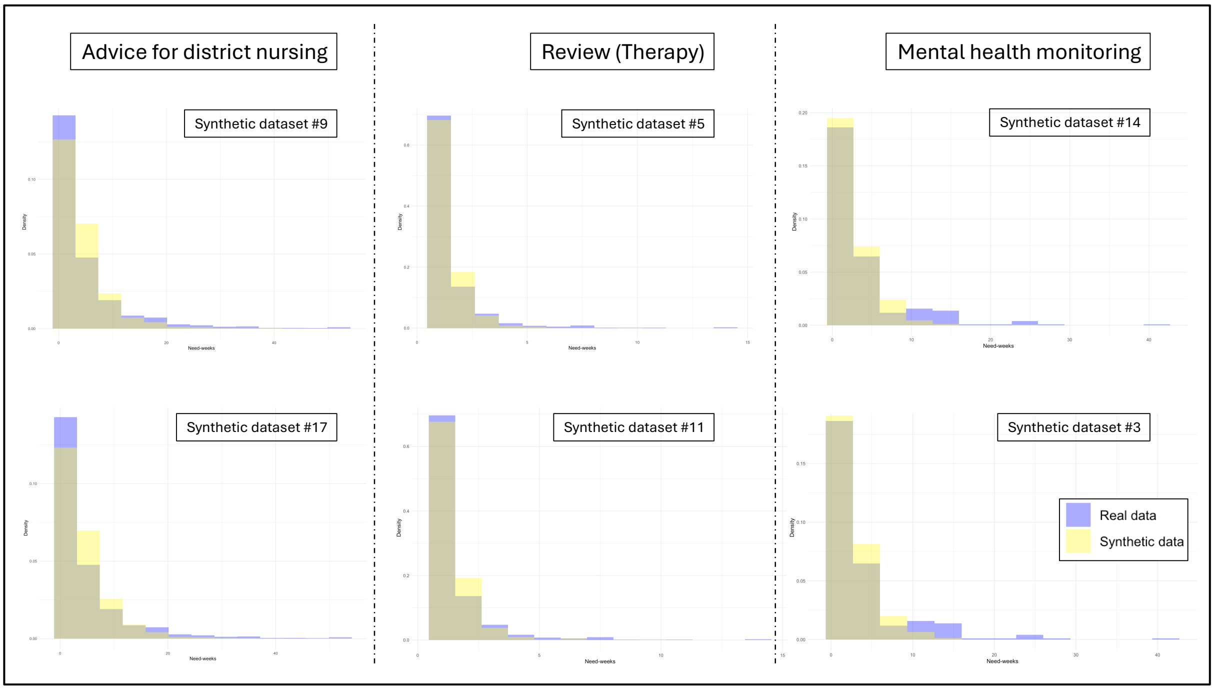

To obtain a quantitative metric measuring the similarity of the real and synthetic distributions, we computed the percent overlap between each pair of distributions (i.e. real vs synthetic, for each activity and for each of the 20 synthetic datasets), and then we averaged that over the number of synthetic datasets to obtain an average percent overlap for each activity – see table below.

The overall average percent overlap across all activities, weighted by the extent of real-world demand, is 88.52%.

Activity	Average percent overlap between real and synthetic need-week distributions
District Nursing - Advice for district nursing	91%
District Nursing - Medication	88%
District Nursing - Wound care	91%
District Nursing - Ulcer care	81%
District Nursing - Blood monitoring	89%
Therapy - Rehabilitation	79%
District Nursing - Daily living	98%
District Nursing - Support	88%
District Nursing - Bandaging	80%
District Nursing - Assessment	99%
District Nursing - Catheter care	73%
District Nursing - Treatment	86%
District Nursing - Diabetic care	86%
District Nursing - Palliative care	89%
Mental Health - Mental health monitoring	90%
District Nursing - Observations	95%
District Nursing - Equipment assessment	90%
Therapy - Review	98%
District Nursing - Post operative care	98%
Therapy - Advice for physiotherapy	98%
Therapy - Physiotherapy assessment	100%
District Nursing - Liaison	98%
Mental Health - Medication	65%
Therapy - Occupational therapy assessment	97%
Mental Health - Memory assessment	100%
Mental Health - Mental health assessment	99%
District Nursing - Bladder care	91%
District Nursing - Prevention of admission	87%
District Nursing - Health promotion	98%
District Nursing - Reassessment	99%
Mental Health - Risk management	91%
Mental Health - Reassessment	99%
Mental Health - Carer support	86%
District Nursing - Continence assessment	98%
District Nursing - End of life care	100%
District Nursing - Bowel care	97%
District Nursing - Doppler assessment	99%
District Nursing - Feeding management	78%
District Nursing - Treatment programme	100%

Activity	Average percent overlap between real and synthetic need-week distributions
Mental Health - Physical health checks	84%
Mental Health - Care coordination	82%
District Nursing - Supervision of medication	96%
District Nursing - Central line care	89%
District Nursing - Risk assessment	99%
Mental Health - Independence	97%
Therapy - Screening	100%
Mental Health - Anxiety management	78%
Mental Health - Psychoeducation	80%
Mental Health - Build confidence	79%
District Nursing - Nutritional advice	99%
District Nursing - Discharge planning	100%
District Nursing - Carer support	100%
Mental Health - Care staff support	77%
District Nursing - Continence advice	98%
District Nursing - Bereavement support	100%
Therapy - Mobility therapy	99%
District Nursing - Pulmonary rehabilitation	75%
Mental Health - Adaptations	99%
Mental Health - Accomodation	94%
District Nursing - Patient training	100%
Mental Health - Relapse prevention	92%
Mental Health - Care programme	100%
Mental Health - Social inclusion	99%
District Nursing - Hydration	100%
District Nursing - Gastrostomy	90%
Mental Health - Enhance potentials	91%
District Nursing - Accident prevention	100%
Mental Health - Falls prevention	100%
District Nursing - Mobility assistance	90%
District Nursing - Chemotherapy	80%
Therapy - Physio discharge planning	100%
Mental Health - Nutritional advice	100%
District Nursing - Tracheostomy care	61%
Therapy - Balance therapy	96%
District Nursing - Safeguarding adults	100%
District Nursing - Funeral attendance	100%
Mental Health - Safeguarding adults	100%

2.2.3 Checking the correlation on combinations of activities

We computed the annual frequencies of the different combinations of activities delivered to patients in the real dataset (i.e. how many patients were delivered each combination of activities). We then computed pairwise correlations (Pearson's correlation coefficient) between the real and synthetic frequencies of activity combinations.

The figure below reports the correlations between activity combination frequencies across each pair of datasets ("real" = real dataset; "s1" to "s20" are the synthetic datasets). The correlation of frequencies in the real dataset versus each of the synthetic datasets range between 0.77 and 0.82.

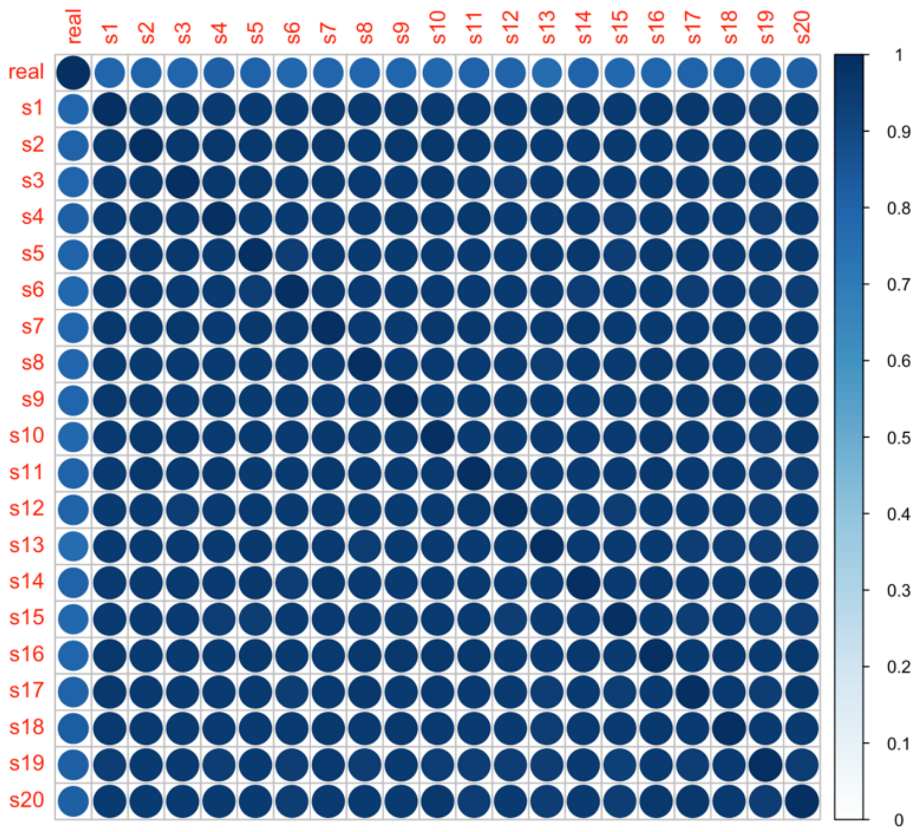

References

1. Grieco L, Utley M, Crowe S. A modelling approach for the analysis of decisions in Home Health Care at multiple planning levels. Health Systems and Quality Improvement; 2025. Available from: http://medrxiv.org/lookup/doi/10.1101/2025.11.06.25339521
2. Dugošija D, Savić A, Maksimović Z. A new integer linear programming formulation for the problem of political districting. Ann Oper Res. 2020 May;288(1):247–63
3. Rodriguez C, Garaix T, Xie X, Augusto V. Staff dimensioning in homecare services with uncertain demands. International Journal of Production Research. 2015 Dec 17;53(24):7396–410
