# Supplementary material for: A mixed methods approach for the identification and assessment of workforce innovations in home health care
Source: Front Health Serv. 2026 Jul 16;6:1749947. doi: 10.3389/frhs.2026.1749947 (PMC13422396; doi:10.3389/frhs.2026.1749947)
Supplement: Supplementary File S3 — CATWOES and root definitions. [file Datasheet3.docx]

# **Healthcare provider organisational perspective (district nursing services)**

**CATWOE**

Customers:

- Adults (>18) who have: a physical healthcare need that would benefit from nursing and who are housebound or have another reason why a home visit is more appropriate (e.g. palliative care)
- Informal carers (e.g. family members) of patients receiving the support
- Health and care professionals who also care for the same patient or would need to care for them in the absence of the nursing support (e.g. social workers, care workers, mental health professionals, health professionals in the acute sector or in cross-Borough community services)

Actors:

- District nurses
- Healthcare assistants / support workers (shared with the therapies team)
- Health and well-being coordinators (non-clinical staff who deliver a time-limited service primarily in the home, typically for socially isolated patients, to free up nursing time spent on non-medical things)

Transformation process:

- Adults with an identified nursing need (including chronic and acute conditions) who are housebound or have another reason why a home visit is more appropriate (e.g. terminal illness) receive nursing care in their own home that enables them to manage their chronic condition or resolve an acute condition, or to be supported palliatively at the end of their life

Worldview:

- Home visits from a district nurse are required for adults who are housebound in order to help them manage their chronic condition or regain independence following an acute illness, and are also the most appropriate way of providing palliative support for adults at the end of their life

Owners:

- Commissioners can withdraw, reduce or increase funding and also change the nature of the activity commissioned
- Referrals are made via GPs and health and social care professionals, and self-referrals can be made by patients

Environmental constraints:

- Commissioners set service contracts that fall short of actual demand and as the services don't turn away eligible patients they do ~25-35% more activity than they are commissioned (and paid) to deliver
- The Borough manifesto sets shared targets and aspirations for the next 20 years and the steering group (incl. GPs, Healthwatch, commissioners, local authority) made the decision to move to place-based care, which underpinned the restructuring of community health services from 6 to 3 localities
- Challenges with staff retention and recruitment mean that some teams are understaffed and rely on bank and agency staff
- Cutbacks in some central corporate services like HR mean that local teams take on the work and have less time to manage the delivery of care
- The central organisation’s drive to reduce its property portfolio impacts where staff are based and how they work with colleagues and with patients
- Central management can define some aspects of service operation such as requiring Agile working

**Root Definition**

Supporting housebound adults who have an identified nursing need including the chronically sick, terminally ill, or acutely unwell BY providing nursing care in their own homes IN ORDER TO help them manage a chronic condition (incl. end of life) and/or recover from acute illness

# **Health professional perspective (district nurses)**

**CATWOE**

Customers:

- Housebound patients with a long-term condition or acute need requiring nursing support
- GPs with patients that they want district nurses to visit, sometimes because they are unable to do a home visit themselves
- Hospitals that need to discharge a patient and want to make sure that the patient is cared for when they return home

Actors:

- Senior district nurses, e.g. who schedule visits and manage the team
- District nurses
- Healthcare assistants / support workers (shared with the therapies team)
- Health and well-being coordinators

Transformation process:

- Housebound patients referred to the service with a nursing need have these needs met and so regain independence or are better able to manage their on-going condition

Worldview:

- Housebound patients with a nursing need will recover or manage their condition most effectively through a package of care delivered by trained nurses in home visits
- It is important for nurses to maintain their professional skills and have the opportunity to specialise so as to be able to provide the best possible care for patients and progress in their career

Owners:

- Commissioners determine the level of activity that nursing teams are supposed to deliver
- The provider organisation sets the service referral criteria but GPs and hospital discharge services also determine who and how many are referred to the service

Environmental constraints:

- Home visits can mean a lot of travelling between patients and patient notes are required for each visit, both of which can limit the time available for face-to-face care
- There are often more patients to see and admin to undertake than is possible within normal hours so overtime and over-working is common
- The length of visits and travel times can be unpredictable, and emergency cases come up, so sticking to specific visit slots for each patient would be challenging
- Variability in the amount and nature of patient demand in different localities, and differences in team sizes, can mean that staff in different teams have different workloads, and it is also challenging to allocate work fairly within teams
- Central management make decisions that affect ways of working, from how teams are split across localities through to Agile working
- The professional body for nursing determines the skill profiles of staff and nurses respond to the code of their professional body
- NHS national pay scales and working conditions influence recruitment and retention
- The strong professional identity of nurses means that they may resist taking on activity traditionally undertaken by mental health nurses, therapists or other professionals

**Root Definition**

Caring for patients referred to our service and developing our professional skills BY scheduling home visits according to the needs of patients and the skills and availability of the team, carrying out nursing tasks that we are qualified in with professionalism and compassion, and undertaking relevant training IN ORDER TO support patients to get better, regain their independence and/or manage a long-term condition.

# **Patient perspective**

**CATWOE**

Customers:

- Residents of the Borough who have a physical health care need requiring nursing support and are housebound or otherwise need the support delivered in their home, who may also have other physical and mental health care problems

Actors:

- District nurses
- Healthcare assistants / support workers
- Health and well-being coordinators
- Physios
- Occupational therapists
- Mental health nurses
- Psychologists

Transformation process:

- A homebound person suffering from a physical health problem requiring nursing and possibly other physical and mental health problems, either acutely or long term, receives the joined up care and support in their home needed to regain independence and carry out activities important to them

Worldview:

- In order for someone to recover and regain independence when acutely unwell they need sufficient face-to-face time with an appropriately trained health professional who focuses on the particular needs of the person
- People have a right to timely, free health care when they need it
- People feel better supported when they are able to establish a relationship with the person/people caring for them, and when they feel that all aspects of their needs and care for in a joined-up way

Owners:

- Tax-payers
- GPs or others that make referrals

Environmental constraints:

- Patients or their informal carers are left to co-ordinate between the different services they receive and professionals that care for them (the system lacks coordination)
- People aren’t always aware of what services are available to them
- Resources for the NHS are constrained, which can mean delays in getting care or hurried visits
- Not everyone is online or IT-literate, which can make it harder to interact with services (human contact is really important)

**Root Definition**

Free healthcare support provided in my home when I need it, such as when I’m acutely unwell, have recently left hospital, or need help with a long-term physical or mental health condition BY trained health professionals who I get to know and trust giving face-to-face care focused on my particular needs IN ORDER TO help me recover and regain my independence, or to help me manage my on-going condition so that I can carry out as many of the activities I want to do as possible.

# **Healthcare commissioning perspective**

**CATWOE**

Customers:

- NHS community, mental health and secondary care providers in a number of London Boroughs
- Primary care providers in a number of London Boroughs
- The residents of the Boroughs
- Local authorities in the Boroughs
- Taxpayers

Actors:

- The commissioning groups are led by elected governing bodies made up of GPs, other clinicians and lay members
- GP practices are key membership bodies, but GPs don't typically take a strategic role in the commissioning cycle in terms of planning, procurement, monitoring and evaluation
- NHS England (and NHS London) who set national (and regional) performance targets that strongly influence commissioning priorities
- The locality Clinical Support Unit, an independent NHS organisation that provides expert contracting support and technical services around IT and data insights to commissioners (incl. for the contract for community and mental health services)

Transformation process:

- The health needs of the local population are met and their preventable health needs avoided through safe, sustainable and high-quality services that are within budget

Worldview:

- Clinicians (particularly GPs) are best placed to assess the health needs of the local population and determine how the local NHS budget should be spent to meet those needs
- Providing preventative services that reduce the need for healthcare can be a sustainable way to optimise the health of the local population using the limited budget
- A considerable amount of activity currently undertaken in acute settings (e.g. geriatric non-elective care and management of long term conditions) could be provided more effectively and cheaply by primary and community care services
- Innovation can help to improve the productivity and quality of services

Owners:

- Clinical commissioning groups are accountable to the Secretary of State for Health and Social Care through NHS England

Environmental constraints:

- Working under a financial recovery plan so savings and productivity gains are needed.
- Ambitions to bring outcomes (e.g. healthy life-expectancy, avoidable admissions, re-admissions) in line with comparable systems within 5 years
- Community health services are commissioned through a block contract with targets on the number of contacts, and it would be challenging to move to commissioning by episodes of care and rewarding services based on patient experience and outcomes
- Contracting and commissioning structures are fragmented and do not enable or support integrated working across services (barriers to integrated working include no IT integration and cultural differences between organisations and how they see each other’s roles).
- There are significant challenges with recruiting and retaining staff, and high use of bank/agency staff
- Ambitions for more joined up services delivered through a locality model of care, facilitated through joint commissioning with the local authorities, capitated outcome-based budgeting and budget-pooling to more appropriately allocate funding to primary and community care and incentivise early intervention and rapid discharge
- In the future, new integrated health and social care structures may influence commissioning and impact on IT and estates
- Funding cuts mean that the capacity to deliver support through the third sector has massively reduced and many third sector services have been de-commissioned

**Root Definition**

A **clinically-led statutory body responsible for planning and c**ommissioning safe, sustainable, high quality services BY working in partnership with the entire health and care community to assess local needs, decide priorities and buy services using the local NHS budget, with a focus on reducing the need for healthcare, innovating, improving quality and increasing productivity IN ORDER TO provide a first-class health service for residents that delivers a better experience and improved outcomes, and optimises the health and healthy life-expectancy of the local population
