# Supplementary material for: A mixed methods approach for the identification and assessment of workforce innovations in home health care
Source: Front Health Serv. 2026 Jul 16;6:1749947. doi: 10.3389/frhs.2026.1749947 (PMC13422396; doi:10.3389/frhs.2026.1749947)
Supplement: Supplementary File S5 — Candidate innovations that emerged through the soft systems analysis. [file Datasheet5.docx]

The candidate innovations that emerged through the soft systems work are listed below, along with their descriptions as well as the stakeholder groups that promoted them.

| **Innovation title** | **Description** | **Stakeholder group promoting innovation** |
| --- | --- | --- |
| Grouping wards into more compact districts to reduce anticipated travel times for staff | The intent of this innovation, introduced to the SSM process from the OR literature, is to reduce the time spent travelling by home-care staff. It aims to do so by using an algorithm to divide the territory of interest into a fixed number of "districts" in a way that gives the most compact districts, minimising the aggregate journey distances between demand centres within a district. | None |
| Grouping wards into districts in a way that balances anticipated workload across districts | The issue of some district-level teams having higher caseloads than others came up in several of the SSM discussions. This innovation, introduced from the OR literature, has the intent of balancing caseload across teams by dividing the territory of interest into a fixed number of districts using an algorithm that minimises the difference between districts of the aggregate caseload from all points of demand within each district. | - Service managers (health and social care) |
| Place-based care - grouping wards into districts that have similar sized populations and borders aligned with political entities and primary care catchment areas | This was the focus of a recent, major restructuring in one of the Boroughs we worked with and was discussed during the SSM process. The intent of this innovation is to improve integration and coordination of health (and social care) services. The rationale is that this can be achieved by bringing organisations together around the population they serve, identifying distinct, geographically defined communities of 50-70 thousand people with common catchment areas across different community health services, and these aligned to political boundaries and catchment areas for, say, primary care services. | - Service managers (health) - Domiciliary care managers |
| Reducing repetition of tasks by several staff working with the same patient | The problem of staff duplicating tasks with a patient already performed by other staff within the same (or more commonly) another team came up in several of the SSM discussions. The intent of innovations to reduce duplication would be to make more efficient use of clinician’s time through improved coordination of care, e.g. by avoiding multiple overlapping assessments of patients. | - Service managers (health) - Service managers (health and social care) - Mixed workshop (health and social care managers and professionals) - Commissioning (social care) - Domiciliary care managers |
| Development of joint health and social care plans for patients | The intent of this innovation would be to improve integration and coordination of care through the development of single health and social care plans for individuals rather than each health care team and domiciliary care team having its own plan for an individual. | - Service managers (health) - Commissioning (health) - Commissioning (social care) |
| Making explicit and extending the roles of informal carers | This innovation is intended to improve the efficient use of staff by harnessing and formalising the role that informal carers play in an individual's care, e.g. in monitoring pressure areas. | - Service managers (health and social care) - Health care professionals - Domiciliary care managers - Informal carers |
| Enhanced care workers - training domiciliary care workers in simple nursing tasks | The intent of ‘enhanced care workers' is to alleviate pressure on NHS services by training care workers to undertake low-level health care tasks with oversight from district nurses. | - Service managers (health and social care) - Health care professionals - Social care professionals - Commissioning (social care) - Domiciliary care managers - Patients |
| Enablement champions - training domiciliary care workers in supporting work of therapy teams | The intent of 'enablement champions' is to alleviate pressure on NHS services and potentially improve reablement outcomes by training some care workers to work with allied health professionals and support patients in doing exercises they have been set by physios for example. | - Health care professionals |
| Greater flexibility in who does what and a shift to more generalist roles | A number of the innovations that were mentioned or proposed during the SSM process were characterised as involving more flexible and/or generalist roles. The intent of such innovation is broadly to reduce the number of visits required to deliver a set of activities by clinicians working across their full range of competencies (or extended competencies), including across traditional disciplinary boundaries in some circumstances. Examples included community matrons changing dressings when visiting a patient to avoid a nurse visiting specially to do so later that day/week, and health professionals across disciplines undertaking pressure area checks and routine observations and performing basic mental health and OT assessments. | - Service managers (health) - Service managers (health and social care) - Health care professionals - Commissioning (social care) - Informal carers |
| Widening accreditation for assessing the need for and ordering of equipment for patients' homes | The intent of this innovation is to obviate a specific bottleneck experienced in the process of occupational therapists ordering the delivery and installation of equipment in patients' homes. It aims to do this through an accreditation scheme that would enable a wider group of staff (including district nurses and allied health professionals) to make such orders. | - Service managers (health and social care) - Commissioning (social care) |
| Introducing the role of a nursing and therapy support worker | This innovation is intended to make more efficient use of clinicians' time by providing an assistant role, and to do so in a way that is efficient and resilient to fluctuations in demand, by creating a role for health care assistants/support workers who are trained to support both nurses and therapists. | - Service managers (health) |
| Introducing a nursing role that covers both mental and physical health | This innovation is intended to reduce the number of visits required by patients and/or create a more flexible workforce through the creation of a nursing role that covers both mental and physical health. | - Service managers (health) - Health care professionals |
| Increased specialisation with staff working predominantly at the upper end of their competencies | Increased specialisation, whereby clinicians spend more of their time on activities at the upper end of their competencies and within hard disciplinary boundaries, was not mentioned in the SSM process as an innovation, but rather as the recent direction of travel. | - Service managers (health) |
| Team size and composition chosen to meet demand at lowest cost | The intent of this innovation is to meet demand at low cost by choosing the number of staff within each role to give a team size and composition that can accommodate a range of plausible patterns of demand at minimum salary costs. | - Service managers (health) - Health care professionals |
| Team size and composition chosen to promote workload balance across staff | The intent of this innovation, introduced to the SSM process from the OR team, is to meet demand without having large disparities in the workload falling on individual team members by choosing the number of staff within each role such that plausible patterns of demand can be met in a way that balances workload. | - Service managers (health) |
| Use of patient acuity scores to promote workload balance within teams | This innovation intends to promote workload balance within teams by using an acuity scoring system to assign points to different patient-facing activities (reflecting complexity of patient need and, for instance, initial assessment versus follow-up) so that when staff are being allocated to patient-visits, this is done with a target "points total" in mind, acting to promote workload balance. | - Service managers (health) - Service managers (health and social care) - Health care professionals |
| Each patient having a named key worker as first point of contact with oversight of care from multiple teams | The intent of this innovation is to improve the coordination of care and, by dint of that, patient experience. The innovation is that, for patients that receive care from multiple disciplines, one of the health care professionals that works with the patient is given the additional role of "care coordinator" (or 'key worker'). This role involves having oversight of the care for that patient, spotting lapses in provision, potential redundancies in the activities of the different teams and opportunities for improved coordination and acting as a single point of contact for the patient. A similar care coordination role could be carried out by dedicated 'support workers'. | - Service managers (health and social care) - Mixed workshop (health and social care managers and professionals) - Commissioning (social care) - Domiciliary care managers - Patients |
| Coordination of home-visits across different teams within health service and across health services and domiciliary care | This innovation would involve coordinating visits between different professionals, potentially across health and social care, with the explicit intent of improving the coordination of care delivered to patients. This could be to avoid a health professional visiting when a care worker is there and, for example, bathing the patient, or to deliberately visit at the same time as care worker so that they can show them how to support the patient in their rehab, or for two or more health professionals to synchronise visits to deliver two-handed tasks or to deliver a shared care plan. | - Service managers (health) - Service managers (health and social care) - Health care professionals - Social care professionals - Mixed workshop (health and social care managers and professionals) |
| Goal-driven allocation of staff to visits, scheduling of visits and routing of staff between visits | This innovation would be to construct (supported by an algorithm or otherwise) allocations of staff to visits, schedules and, sometimes, routes with the aim of either:  - increasing the chance of punctual visits and lowering the chance of visits being cancelled at late notice (by accounting for variation in visit durations), or - promoting continuity of relationships, or - minimising differences in workload within a team or, by incorporating some allocations across district boundaries, across the workforce as a whole, or - reducing cost / number of visits, or - minimising the time spent travelling | - Service managers (health) - Service managers (health and social care) - Health care professionals |
| Promotion of "agile working" through for example increased staff use of portable devices | Agile working is the name given to an innovation within the service that has enabled staff to minimise travelling back to the base in between appointments (e.g. to write patient notes), predominantly through the use of portable devices. | - Service managers (health) - Service managers (health and social care) - Health care professionals - Social care professionals |
| Improved quantitative assessment of population needs across services and teams | This innovation is intended to promote equity of provision by rooting commissioning decisions in analysis of met and unmet need and addressing historic differences in levels of provision across the territory. | - Service managers (health) - Commissioning (health) |
| Active, goal-driven queue management | This innovation is intended to improve outcomes by managing waiting lists and monitoring the condition of patients while they are waiting for services to identify and then prioritise patients whose health may deteriorate to the point where they need a higher level of service when they are seen. | - Health care professionals |
| Increased focus on early intervention and prevention | This innovation, which came up in several discussions during the SSM, involves a shift in emphasis towards prevention and earlier intervention. Rather than being a single specific innovation, this would involve revising services and a phased rebalancing of resources within and between sectors to increasingly focus on early interventions that have the potential to reduce / delay demand for more resource-intensive health and/or social care. | - Service managers (health) - Service managers (health and social care) - Health care professionals - Commissioning (health) - Commissioning (social care) - Community-based assets |
| Offering patients choice of visit times | This innovation, intended to make care more patient centred and improve patient experience, would be to grant patients a degree of choice on when visits from health professionals are scheduled. | None |
| Shared access to electronic health and social care records | This innovation would entail improvements to IT systems to grant all health professionals and/or social workers involved in the care of a patient access to that patient's notes from across different teams and services. | - Service managers (health) - Service managers (health and social care) - Health care professionals - Social care professionals - Mixed workshop (health and social care managers and professionals) - Commissioning (social care) |
| Messaging patients when staff running late | This innovation would entail an automated system whereby patients would be alerted (for instance by text) if the health professional due to visit them was running late. | None |
| Joint commissioning of health and social care from a pooled budget | This innovation would involve moving to a shared budget and joint commissioning between health and social care to support better integration and coordination of health and social care, and to enable joint health and social care plans for patients. | - Service managers (health) - Health care professionals - Commissioning (health) - Domiciliary care managers |
| Use of advanced computational algorithms to support rostering | The intent of this innovation, introduced to the SSM process from the OR literature, is to increase the resilience of home health services to fluctuations in demand and staff absence by using computational algorithms to support the process of constructing staff rosters. This could also free up some of the senior clinician time currently devoted to rostering and could ease the introduction of more part-time working. | - Service managers (health and social care) |
| Increased use of personal health budgets | Personal health budgets are a way of personalising care, based around what matters to people and their individual strengths and needs. They give disabled people and people with long term conditions more choice, control and flexibility over their health care. A personal health budget may be used for a range of things to meet agreed health and wellbeing outcomes. This can include therapies, personal care and equipment. | - Domiciliary care managers |
| Systematic use of social prescribing and signposting | This innovation would involve systematic use of social prescribing and signposting to enable people to gain the support they need outside of the health and care system so as to reduce current and future demand on services. Includes 'care navigators'. | - Service managers (health and social care) - Social care professionals - Commissioning (social care) - Community-based assets |
| Personalisation of social care plans | This innovation involves greater flexibility in how social care plans are delivered, encouraging carers to work creatively with clients to find the best way of delivering care for them, addressing the client's needs and social isolation. | - Commissioning (social care) - Domiciliary care managers |
| Outcomes-based commissioning | This innovation is intended to improve outcomes by moving away from commissioning specified volumes of activities and instead allowing providers to identify the most effective and efficient ways to working that deliver a set of specified outcomes, with outcome data becoming the focus of contractual management. | - Commissioning (health) - Commissioning (social care) - Domiciliary care managers |
| Trusted assessors - training domiciliary care workers to assess evolving needs of clients | This innovation involves training some domiciliary care workers to become Trusted Assessors that are able to assess the changing needs of clients and liaise with social workers to amend care plans accordingly. | - Social care professionals - Commissioning (social care) - Domiciliary care managers |
| Increased use of personal care budgets | This innovation is to increase the use of Personal Care Budgets and promote the use of Personal Assistants with the intent of enhancing the personalisation of care. | - Commissioning (social care) - Domiciliary care managers - Patients |
| The "three conversations model" of needs assessment, early intervention and drawing on personal strengths and community assets | The three conversations model for social care, which was discussed by several stakeholders, is an approach to needs assessment and care planning focusing primarily on people's strengths and community assets with the aim of sustaining a person's independence. The innovation has elements of early intervention and, more broadly, looks to minimise unnecessary statutory care provision. | - Service managers (health and social care) - Social care professionals - Commissioning (social care) |
| Virtual clinics and telehealth | Virtual clinics, for appointments that don't require in-person care, and telehealth used for remote monitoring, for example, are innovations aimed at reducing the number of face-to-face visits, saving travelling time for health professionals. | - Service managers (health and social care) - Domiciliary care managers |
| Transporting home-bound patients to clinics | This innovation, which was mentioned as a potential way to treat more patients with limited resources, would involve transporting homebound patients to and from day centres where they could receive the care they need in a clinic, saving travelling time for health professionals. In mental health it is best practice to do home visits (particularly for assessments), but the community healthcare provider was starting to think whether some of these could be done in clinics as they are so short staffed. | - Service managers (health) - Service managers (health and social care) - Health care professionals |
| Introducing non-clinical roles to support reablement and address social isolation | This innovation came up in several discussions during the SSM, with example roles including 'health and well-being coordinators' who support socially isolated patients to free up nursing time, ‘support time recovery workers’ who provide companionship and help to resolve practical issues for people with mental health conditions to support recovery and independent living, and 'reablement support workers' who support patients to regain skills and confidence so that they can do everyday tasks and activities themselves safely. | - Service managers (health) - Service managers (health and social care) - Health care professionals - Commissioning (social care) - Community-based assets |
