# Supplementary material for: A mixed methods approach for the identification and assessment of workforce innovations in home health care
Source: Front Health Serv. 2026 Jul 16;6:1749947. doi: 10.3389/frhs.2026.1749947 (PMC13422396; doi:10.3389/frhs.2026.1749947)
Supplement: Supplementary File S6 — Mapping of candidate innovation to motivating aspects of system performance. [file Datasheet6.docx]

Mapping of each candidate innovation to one or more motivating aspect of system performance. We also indicate whether the innovation could be evaluated using the quantitative modelling framework

| **Innovation title** | **Aspects of system performance (the driving motivation)** | **Model** | **Illustrative analysis in paper?** |
| --- | --- | --- | --- |
| Grouping wards into more compact districts to reduce anticipated travel times for staff | - How the time of clinical staff is spent | - Districting |  |
| Grouping wards into districts in a way that balances anticipated workload across districts | - The sustainability of the workforce | - Districting | Innovation 3 |
| Place-based care - grouping wards into districts that have similar sized populations and borders aligned with political entities and primary care catchment areas | - Integration and coordination of care - Equity of provision |  |  |
| Reducing repetition of tasks by several staff working with the same patient | - How the time of clinical staff is spent - Integration and coordination of care - Continuity of information | - Workforce roles & Home health care package |  |
| Development of joint health and social care plans for patients | - Integration and coordination of care |  |  |
| Making explicit and extending the roles of informal carers | - Cognisance and use of other support available to individual patients - How the competencies of staff are deployed - Number of patients treated within budget and to an acceptable standard |  |  |
| Enhanced care workers - training domiciliary care workers in simple nursing tasks | - Integration and coordination of care - The sustainability of the workforce - Staff skills mix - How the competencies of staff are deployed | - Workforce roles & Home health care package |  |
| Enablement champions - training domiciliary care workers in supporting work of therapy teams. | - Integration and coordination of care - The sustainability of the workforce - Staff skills mix - How the competencies of staff are deployed | - Workforce roles & Home health care package - Team size and composition |  |
| Greater flexibility in who does what and a shift to more generalist roles | - Staff skills mix - How the time of clinical staff is spent - How the competencies of staff are deployed - Integration and coordination of care | - Workforce roles & Home health care package - Team size and composition | Innovation 2 |
| Widening accreditation for assessing the need for and ordering of equipment for patients' homes. | - How the time of clinical staff is spent - How the competencies of staff are deployed - Times between referral, assessment and access to services |  |  |
| Introducing the role of a nursing and therapy support worker | - Staff skills mix - How the competencies of staff are deployed | - Workforce roles & Home health care package - Team size and composition |  |
| Introducing a nursing role that covers both mental and physical health | - Staff skills mix - How the competencies of staff are deployed | - Workforce roles & Home health care package - Team size and composition |  |
| Increased specialisation with staff working predominantly at the upper end of their competencies | - How the competencies of staff are deployed | - Workforce roles & Home health care package | Innovation 1 |
| Team size and composition chosen to meet demand at lowest cost | - Staff skills mix - The number of patients treated within budget and to an acceptable standard | - Team size and composition |  |
| Team size and composition chosen to promote workload balance across staff | - The sustainability of the workforce | - Team size and composition | Innovation 4 |
| Use of patient acuity scores to promote workload balance within teams | - The sustainability of the workforce | - Allocation and scheduling |  |
| Each patient having a named key worker as first point of contact with oversight of care from multiple teams | - Integration and coordination of care - Patient experience - Patient centredness - Continuity of relationships - Continuity of information |  |  |
| Coordination of home-visits across different teams within health service and across health services and domiciliary care | - Integration and coordination of care |  |  |
| Goal-driven allocation of staff to visits, scheduling of visits and routing of staff between visits | - Punctuality of home visits - Cancellations and postponements - Continuity of relationships - The sustainability of the workforce - The number of patients treated within budget and to an acceptable standard - How the time of clinical staff is spent | - Allocation and scheduling (Routing not included) | Innovations 5,6,7,8 |
| Promotion of "agile working" through for example increased staff use of portable devices | - How the time of clinical staff is spent |  |  |
| Improved quantitative assessment of population needs across services and teams | - Equity of provision |  |  |
| Active, goal-driven queue management | - Times between referral, assessment, and access to services - Longer-term impacts - Outcomes and costs outside of the home care sector |  |  |
| Increased focus on early intervention and prevention | - Longer-term impacts - Outcomes and costs outside of home care sector |  |  |
| Offering patients choice of visit times | - Patients scheduled to be seen at times convenient to them - Patient experience |  |  |
| Shared access to electronic health and social care records | - Continuity of information - Integration and coordination |  |  |
| Messaging patients when staff running late | - Patient experience |  |  |
| Joint commissioning of health and social care from a pooled budget | - Integration and coordination of care |  |  |
| Use of advanced computational algorithms to support rostering | - The sustainability of the workforce - How the time of clinical staff is spent |  |  |
| Increased use of personal health budgets | - Degree of personalisation |  |  |
| Systematic use of social prescribing and signposting | - Cognisance and use of other support available to individual patients |  |  |
| Personalisation of social care plans | - Degree of personalisation - The safety and efficacy of services / treatments |  |  |
| Outcomes-based commissioning | - The safety and efficacy of services / treatments - Outcomes and costs outside of home care sector - Longer-term impacts |  |  |
| Trusted assessors - training domiciliary care workers to assess evolving needs of clients | - The timing and accuracy of reassessments |  |  |
| Increased use of personal care budgets | - Degree of personalisation |  |  |
| The "three conversations model" of needs assessment, early intervention and drawing on personal strengths and community assets | - Cognisance and use of other support available to individual patients - Degree of personalisation - Longer-term impacts - The number of patients treated within budget and to an acceptable standard |  |  |
| Virtual clinics and telehealth | - The number of patients treated within budget and to an acceptable standard - How the time of clinical staff is spent |  |  |
| Transporting home-bound patients to clinics | - The number of patients treated within budget and to an acceptable standard - How the time of clinical staff is spent |  |  |
| Introducing non-clinical roles to support reablement and address social isolation | - Staff skills mix - How the time of clinical staff is spent - Patient experience - Longer-term impacts |  |  |
