# Supplementary material for: A mixed methods approach for the identification and assessment of workforce innovations in home health care
Source: Front Health Serv. 2026 Jul 16;6:1749947. doi: 10.3389/frhs.2026.1749947 (PMC13422396; doi:10.3389/frhs.2026.1749947)
Supplement: Supplementary File S7 — “Readiness for change” checklist. [file Datasheet7.docx]

**Assessing Readiness and Capacity to**

**Undertake Operational Changes in Work Systems**

K.D. Eason and W. Maton-Howarth

Bayswater Institute

June 2021

**1. Developing a Checklist for Readiness to Change**

In the early WORKTECC studies in the two London Boroughs it rapidly became apparent that community healthcare services have been subject to many changes in recent years. We examined sixteen recent innovations to identify the factors that defined their readiness to undertake these changes and the capacity they were able to devote to the endeavors. Some of the changes were successfully undertaken, notably changes to organisational structure and the introduction of new technical systems. But others, particularly those that involved changes in work roles and behavioural change proved more difficult to implement.

We used the data about the factors that were important in these change processes to create a checklist of factors that could be used to assess readiness to change. We have subsequently used the checklist as a predictive tool to assess the readiness of one of the Boroughs to implement the innovations identified by the OR modeling in the project.

In sections 2, 3 and 4 of this paper the rationale and composition of the checklist is described. Section 5 presents the structure of the checklist as used for the assessment of readiness to implement future innovations.

The checklist covers both general factors that would apply to any change being made and specific factors that relate to the particular change being planned. An important prerequisite before as assessment can be made of the factors that are important in relation to a specific change is to model both the existing work system that will be impacted (the ‘As Is’ system) and the work system as it would be after the change (the ‘To Be’ system). An understanding of the journey the organisation needs to make between these two system states provides the basis for assessing the changes that will be needed in each part of the existing system. In section 6 the methods used to describe the existing system and the future system are presented.

**2. The Proximal and Distal Systems Perspectives**

If a change is to be introduced into a complex work system in which there are many interdependent relationships there will be issues to address at the overall work system level in addition to the local level where the change has the most direct impact. When systems change is considered using sociotechnical systems theory (Emery and Trist 1965, Eason 2014) a distinction can be made (Roberts and Geels 2019) between the Proximal and the Distal system perspectives where the Proximal perspective is about the system that is closest to the point of the change and the Distal perspective covers the rest of the system that is more distant from the change. Figure 1 uses this distinction to identify the issues to be addressed at these two system levels if a change is to be successfully undertaken.


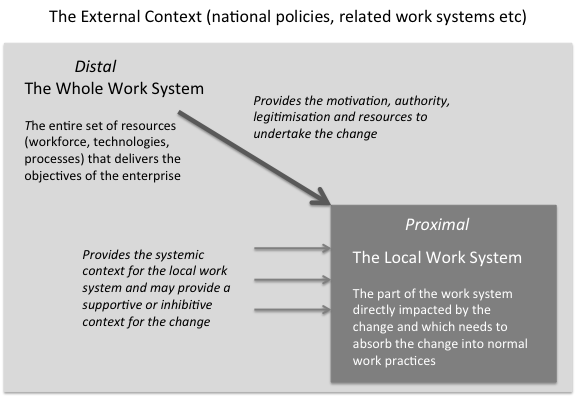


*Figure 1*

*The Proximal and Distal Perspectives*

The Distal perspective includes the whole work system. In WORKTECC it is the whole system in the two boroughs that delivers community healthcare (the workforce, the work processes and the various technologies in use).

The Proximal perspective refers to the local work system that is directly impacted by a specific change and will be different for each change. It might, for example, be a multi-agency local team that is adopting a new social prescription practice or it might be a more wide-ranging change such as the adoption of mobile technology by all the teams delivering front line service. The Proximal perspective is a more detailed examination of a section of the work system that is embedded within the whole system.

Although the Proximal perspective examines in detail the work that needs doing to implement change, this work will be supported or hindered by properties in the whole system covered by the Distal perspective. In its turn, the Distal perspective includes a bigger system perspective because the whole work system operates within the broader context of, for example, government policies, which may also support or hinder specific changes. Another way of depicting these systemic relationships, using the concepts of open systems theory (Katz and Kahn 1966), is to say it consists of a set of nested systems with permeable boundaries.

The whole work system serves two functions with respect to the local work system that is actually delivering a change. First, the whole system includes mechanisms by which authority, responsibility and resources can be distributed and these mechanisms need to provide the wherewithal for the change process to take place: the motivation for it to happen and the authority and legitimization that will enable project staff to proceed. Most importantly it will also need to provide the resources needed to get the work done: these may be human resources from staff roles e.g. I.T. staff, external resources with expertise in the change in question or additional resources for front line staff to enable them to devote time to the change process. The authorization process may also include project management issues such as timescale, reporting requirements and expectations of outputs.

The second function is quite different. The whole system also provides the context within which the change will proceed and it is a context that may both support and inhibit the change. Three elements may be important:

- The prevailing culture of change Is change the norm or is stability and continuity prized? Are some kinds of change easily undertaken whilst others are very difficult to undertake? Is there a culture in some parts of the organisation that embraces change whilst other parts resist it?
- Other change programmes In a large, complex work organisation it is likely that other changes will also be underway. If they are aligned with the planned change they may provide ‘pull through’ or they may be pulling in a different direction and represent obstacles to progress.
- Systemic ramifications Many of the systemic ramifications of a change will be local but there may be others that are wide ranging and deep seated in the entire work system and these could be obstacles to change. For example, changes in work roles may be difficult if current role definitions are based on national qualifications that define professional expectations and responsibilities and these apply across the entire workforce.

The Proximal perspective relates to the local part of the work system that will be most directly impacted by the change being considered. It is in this local part of the work system that the majority of new practices will need to be embedded into the way work is undertaken and the impact of each change at this local level will be examined in detail for specific factors determining readiness for change.

In the next sections, we have used the data about recent changes undertaken in the two Boroughs, and data from other change processes, to list the significant issues from the Distal and Proximal perspectives that determine readiness to change and the capacity of the system to proceed with all the activities necessary to embed a change in a sustained way.

**3. The Distal Perspective**

Figure 2 lists 22 factors associated with the whole work system that could act to support change activities at a local work system level or to hinder it. Section 1 lists the ways in which the change project may be formally launched and supported. One particular set of issues surrounds the last two items: in a lot of cases support is time limited but the processes of embedding a change in normal practice and of spreading it to other parts of the system may take an extended period of time.


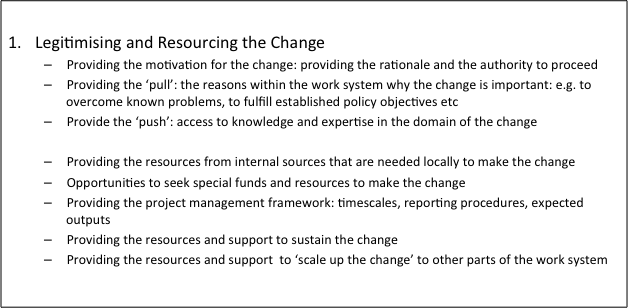


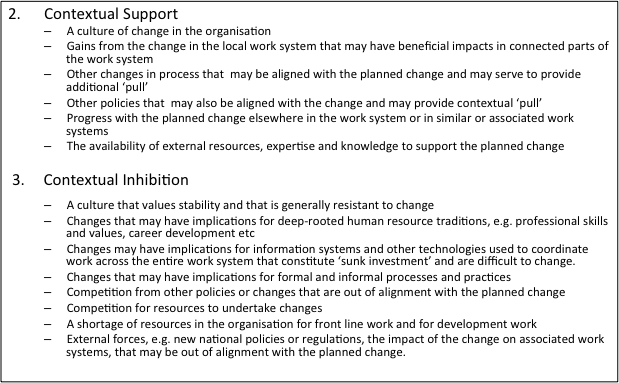


*Figure 2*

*Whole System Factors Influencing Capacity for Change.*

The contextual issues in figure 2 are divided into those that could provide support for a planned change and those that might inhibit it. In some cases they are the mirror issues of one another, e.g. a culture where change is the norm will be a supportive influence, if the emphasis is on sustaining the status quo, the culture will not provide a supportive environment. The factors listed in figure 2 are of several different kinds: (i) cultural norms shared across the whole system, (ii) deep-rooted existing structures, traditions and systems that would be difficult to change if they impeded the incoming change, (iii) the influence of other policies and change programmes being pursued, (iv) the availability of resources to engage in developmental work and (v) the influence of external forces, e.g. new government policies, national programmes for similar changes and progress being made by comparable work systems.

**4. The Proximal Perspective**

As figure 3 below indicates, having the capacity to deliver change in the local work system involves two important activities: (i) creating and operating a project ‘vehicle’ for undertaking the work and (ii) undertaking the systems work to embed the change in the existing local work system. There will also be many change-specific tasks to undertake, for example, transferring data to new information systems, training staff in new practices etc. However, we have not detailed these issues here because we are concerned with the ability of the existing work system to engage with change. The focus in the work to be undertaken is therefore upon the identification of systemic ramifications of the change being considered and the ability of the change programme to deal with these ramifications.


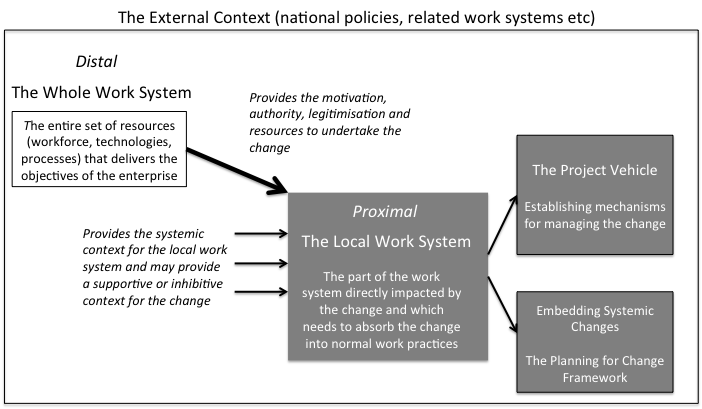


*Figure 3*

*Components of the Local Work System Required to Embed Systemic Change*

In considering changes to a community health system we are dealing with an existing system that delivers services on a daily basis and will need to continue doing so whilst any changes are made. Any change will have a specific target within the overall work system as depicted in Figure 4 and the first priority in assessing readiness to change is the impact the change will have on the components of the target part of the work system.


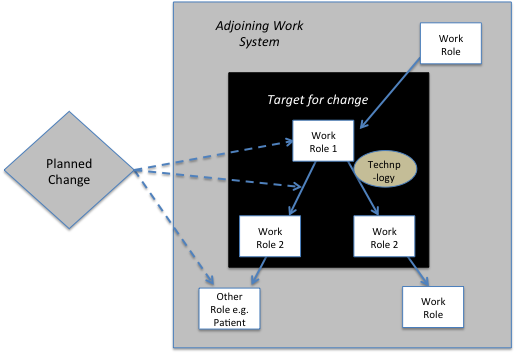


*Figure 4*

*The Impact of the Planned Change on the Local Work System*

Following the systems description methods adopted in sociotechnical systems theory (e.g. Eason et al 1996, Carayon et al 2020), we can ask what will be the impact on the staff in the work roles within the target sub-system, what will be the impact on other roles such as the patient, what will be the impact on the work processes the staff fulfill and what will be the impact on the tools and technologies they use to do the work. Since we are dealing with a work system, it is also necessary to look beyond the direct impact on a particular system element and examine the way in which the change may have indirect impacts through the ‘task interdependencies’ (Herbst 1974) in the system, i.e. the way in which the performance of one task has an impact on other tasks.

The target work system is part of the bigger work system and will have task interdependencies with parts of the system outside the target. It might, for example, be the case that, as a result of a re-organisation of district nursing, GPs find themselves having to adjust to new teams caring for their patients.

Many studies have found that the reasons change is often difficult to accomplish is that there are unforeseen consequences of the change for other parts of the overall system and these ramifications become major obstacles (Eason 2014). In assessing readiness for change it is therefore necessary to extend the detailed analysis of the impact of the change beyond the immediate target and into the adjoining parts of the work system.

In summary, the assessment of readiness to change at the local level where the change impacts specific parts of the work system, examines the direct impact on work roles, work processes and supporting technical systems and extends the assessment to adjoining parts of the work system where there may be secondary impacts. The overall assessment provides a systems account of the work to be done in order for the change to become embedded as normal practice in the work system.

In order to bring about these changes there will need to be a project ‘vehicle’ i.e. a project team with plans and resources to engineer the changes from initial planning through to the sustained embedding of the change in the normal work practices of the enterprise. The features of the project vehicle that determine whether it has the capacity to deliver the change are listed in figure 5. In order to successfully embed the change into the existing working processes and practices in the local work system, the project team will need to examine all the points where the change impacts existing practices.

Figure 5 divides the properties needed in the project vehicle to deliver the change into three phases. Initiation is the stage when the resources are acquired, responsibilities determined and plans laid. The second stage is Engagement because a strong outreach element will be required to engage with both the workforce directly impacted by the change and those in adjoining parts of the work system who may be indirectly impacted. The final set of issues concerns the longer-term issues of embedding and sustaining the change and of scaling-up the change and applying it elsewhere in the whole work system.

The final question in assessing readiness to change returns us to the Distal perspective of the change and in particular to part 1 of figure 2: will the authorities responsible for the entire work system be willing and able to commit the resources necessary to bring about the change having appreciated the facilitating forces and potential barriers elicited by the other parts of the assessment?


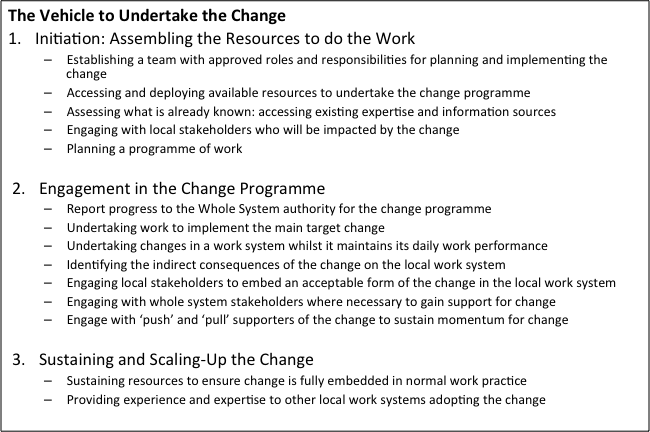


*Figure 5*

*The local project vehicle needed to deliver the change*

**5. The ‘Readiness for Change’ Checklist**

Figures 6, 7, 8 and 9 below present the structure of the checklist used to address the four elements of the assessment of readiness to change described above. It is possible in figures 7, 8 and 9 to specify each topic area being addressed. This is not possible for figure 6 because it assesses the particular impact of the change on specific parts of the local work system and these issues are only revealed through the analysis of the current work system (the ‘As Is’ assessment) and the system as it would be after the planned change (the ‘To Be’ assessment). An example of the ‘As Is’ and ‘To Be’ analysis is provided in section 6.

| Work System  Element | Probable Change | Positive Impact/  Facilitator | Negative or  Problematic Impact/ Potential Barrier |
| --- | --- | --- | --- |
| Work Roles |  |  |  |
| Work Roles Directly Impacted by Change |  |  |  |
| Work Roles  Indirectly Impacted by Change |  |  |  |
| Other Roles |  |  |  |
| e.g. The Patient |  |  |  |
| Technology |  |  |  |
| Information Sources |  |  |  |
| Other Technical Resources |  |  |  |
| Work Processes |  |  |  |
| New Processes/Major Changes |  |  |  |
| Processes Indirectly Impacted |  |  |  |

*Figure 6*

*Assessing the impact of a change on the local work system (Proximal Perspective)*

The checklist examines the impact of the change on three facets of the local work system: the roles of human agents in the work system, the processes by which work is undertaken and the technologies and tools that support the work. The first part of the analysis is to list all of the work roles that would be directly affected by the change and to specify the nature of the change as it would impact each role. It is important to capture where the impact occurs through a task interdependency, e.g. the role of job rostering for district nurses may change for a team manager because it is supported or undertaken by a computer aid but there will then be indirect effects on the district nurse team members who may be asked to different visits than previously.

The second part of the checklist lists the work roles in the adjoining work systems that may be indirectly impacted by the change. It is in the nature of systemic assessment that the further away you move from the main site of the change the more the conjecture is involved in establishing the likelihood and form of a possible impact. Nevertheless, it can be important to identify possible impacts because in many change programmes it has been the indirect ramifications of change that have proved more problematic.

The checklist also recognizes that there will be other roles than those played by healthcare staff that could be impacted by a specific change. Of particular significance in the healthcare domain is the impact of any change on the service delivery to the patient.

The impact upon existing technical systems can also be direct or indirect. If the change involves a new or modified technical system there will be issues relating to the systems it replaces. Many technical systems have their own interdependencies and a change in one can have ramifications for others.

The processes by which work is undertaken can also be subject to major change and again because processes are interdependent, a change in one process can have knock-on effects on other processes.

The checklist seeks to identify all the changes that might occur and then asks whether the specific change will be seen as a facilitating or inhibiting factor in the overall adoption of the change. Ideally, this judgement is best made by staff who are in significant roles in the work system. We have, for example, run workshops in which the specific changes associated with a change programme are identified and local staff asked to evaluate the impact on their roles and work processes (Eason 2007). These workshops had the effect of engaging staff in planning for the change and often began a process of capitalizing on positive impacts and finding ways round potentially inhibiting impacts.

The next section of the checklist is the Distal assessment of the overall context for a change in the entire work system as listed in figure 7.

| **Part 2: Contextual Support and Inhibition (Distal View)** | **Facilitating Factors** | **Inhibiting Factors** |
| --- | --- | --- |
| 1. ‘Pull’ factors that are specific to this innovation. e.g. objectives and policies that the innovation may support |  | N/A |
| 2. Objectives and policies that may inhibit the innovation | N/A |  |
| 3. Other planned changes/recent innovations in the local work system that may impact the innovation |  |  |
| 4. The general culture of change, i.e. progress with similar changes in recent years |  |  |
| 5. The impact of the change on the existing Borough-wide system for delivering community healthcare, including workforce deployment, work processes and technical support |  |  |
| 6. Evidence from similar innovations in other community health teams |  |  |
| 7. Availability of internal/external expertise/resources to support the implementation of the innovation |  |  |

*Figure 7*

*The Overall Context for the Change (Distal Perspective)*

The review of the overall context for change in the work system begins by considering what other changes are planned or are in progress. Some existing policies and plans for change, for example, may align with the specific planned change and may provide a ‘pull through’ context. Other policies and planned changes may, however, have the reverse effect: they may be pulling the organisation in a different direction and may impede progress. Finally, there may be planned changes that are neutral to the new change but could be obstacles because they are competing for resources.

The Distal perspective also examines the overall culture for change that may impact the planned development. Some factors may impact all planned changes. In the current context for example limits on budgets, the growing workload and now the effects of the pandemic means front line staff are stretched to deliver their daily workload and there is very little frontline ‘organisational slack’ (Nohria and Gulati 1997) available to devote to other change programmes. However, the culture for change may also vary depending on the type of change being considered and progress with similar changes in the recent past may provide a good guide to what may happen with a new progamme.

The implementation of a change at the local level may require changes in features of the work system that are also common elsewhere in the work system, e.g. changes to information systems in use elsewhere or changes to HR policies and practices that also apply elsewhere. These kinds of ramifications can have profound consequences for readiness to change because they may involve change across the entire work system.

Finally, progress with a change programme can be influenced by the availability of evidence about similar innovations undertaken elsewhere, not only evidence of the benefits obtained but also evidence of ‘how to do it’. The availability of specialist skills and knowledge relevant to the change both inside the organisation and outside may also be important.

The third section of the checklist is concerned with the project ‘vehicle’ needed to deliver the specific change programme.

| **Part 3: The Local Programme to Deliver the Innovation (Proximal View)** | **Requirement** |
| --- | --- |
| 1. Initiation: Setting up the change programme |  |
| 2. Engagement: The roles in the programme of stakeholders impacted by the change |  |
| 3. Sustaining/Scaling-Up the Change: Ensuring the change becomes embedded as normal practice across the wider system |  |

*Figure 8*

*The Project Vehicle Needed to Deliver the Change*

Although there may be many project management issues to address, the checklist focuses upon what will be needed to tackle the issues identified in section 1 of the checklist, i.e. what changes will need to occur in the target system and in adjoining systems for the change to become embedded as normal practice. In particular, if the development requires significant change in work roles, it may be important to consider how to engage the staff affected in the planning of the change. Another issue is that many change programmes make only limited progress because the project is time limited and, depending upon the changes required in the work system, it may be necessary to sustain the programme over a longer time period to see that changes in working practices are sustained. In many circumstances, changes are first implemented in one part of the work system, and it may be necessary to scale-up the programme so that all parts of the work system can implement the change in due course.

Considering what will be required to progress the change at the Distal level provides information to inform the last part of the checklist: the way the change programme is authorized and resourced. This is likely to be a management decision based upon the business case for the change (the expected benefits) but the conclusions from the checklist about the specific readiness for the change in the organisation and the resources that would be required should also inform the decision. The checklist will show both the factors that support the change, those that might represent obstacles and the particular resources that will be required to make progress.

| **Part 4: Authorising and Resourcing the Innovation (Distal View)** | **Issues** |
| --- | --- |
| 1. What would be the rationale (business case) for authorizing this innovation? |  |
| 2. What would be the countervailing arguments for not authorizing the innovation? |  |
| 3. What resources would be required to ensure the innovation was implemented and became embedded as normal practice? |  |

*Figure 9*

*Authorising and Resourcing the Change*

**6. Representing the ‘As Is’ and ‘To Be’ Work Systems**

Work systems of any scale are complex and defy easy representation. Many different methods are in use that serve an array of different purposes. Formal organisational and pathway depictions may emphasize, for example, how responsibilities are allocated and the official ways in which work is to be undertaken. Soft-system ‘rich pictures’, (Checkland 1981), by contrast, capture the often quite different perspectives on the work system by the different stakeholders in it whilst systems dynamics diagrams (Senge 1990) look at the work flows and possible feedback loops that account for the often counter-intuitive outcomes of changes in work processes. For the purposes of making a systemic assessment of readiness to change in a work system we needed a method of representation that emphasised the interdependence of work roles, work processes and technical systems in the production of work that would facilitate the identification of systemic ramifications of a change and well as its direct impacts. Sociotechnical systems theory has a long history of representing work systems in this way beginning with initial work at the Tavistock Institute of Human Relations (Herbst 1974) and progressing to more formal representations, for example, ORDIT (Organisational Requirements Definition for IT systems) diagrams developed in a European Union ESPRIT project (Eason et al 1996) and the SEIPS (Systems Engineering Initiative for Patient Safety) approach developed by Carayan et al in the USA. Formal representations of sociotechnical systems can be quite complex and for the purposes of assessing readiness for change we have adopted a simplified version and only applied it to the target sub-systems of any change and selected parts of adjoining systems where there are clear task interdependencies.

In figures 9 and 10 below we have used the analysis of one of the innovations explored in WORKTECC (but not part of the OR modeling process) to demonstrate how the ‘As Is’ and ‘To Be’ work systems have been depicted. Our purpose here is not to present a full analysis of readiness for change but to illustrate how the diagrams provide the basis for the completion of the first part of the checklist as shown in figure 6.


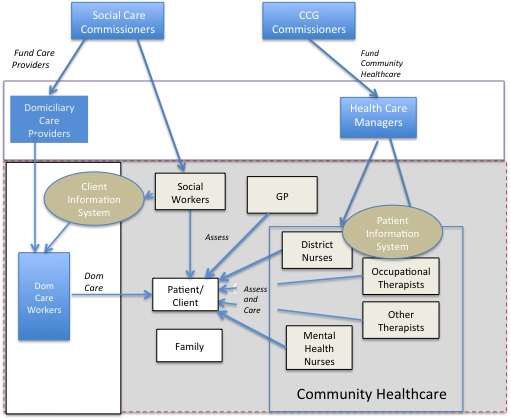


*Figure 10*

*A Sociotechnical Systems Representation of the ‘As Is’ System for Domiciliary Care*

One possible innovation which would help hard pressed community healthcare staff would be to enhance the work of domiciliary care workers so that in their regular visits to clients they also undertake some of the more routine healthcare tasks that are normally undertaken by healthcare staff. Figure 10 is an outline description of the ‘As Is’ relevant part of the community health and social care work system that might be impacted by such a development. It includes the roles of domiciliary care workers, their managers and the social workers who assign work to domiciliary care. It obviously includes the patients for whom the care is provided and all of the healthcare staff who may be providing care for the patients including their GP practice and the nurses and therapists in the community healthcare team. Some of the most important work processes are depicted in the diagram as are the most important technical systems, the client information systems kept for social care and the patient information systems kept for patient care.

Figure 11 inserts the innovation of ‘Enhanced Care Workers’ into the target work system that is domiciliary care and shows the main ramifications that would produce changes elsewhere in the adjoining work systems.


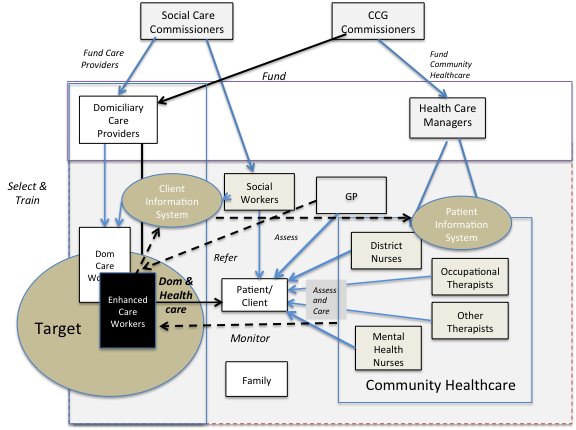


*Figure 11*

*The ‘To Be’ System After the Creation of Enhanced Care Workers Delivering Health and Social Care*

The main change would be that the ‘Enhanced Care Workers’ are now providing some healthcare to their clients but to do this requires other changes. The direct changes (solid lack lines) are that care workers would need to be selected and trained for their extended role and funding from CCG commissioners would need to be agreed. The indirect changes (dotted black lines) are that specific patients would need to be referred, probably by their GP, and the work undertaken would need to be reported to the relevant healthcare workers so they take account of it in their own work. One part of the reporting could be that results of the work would be logged in the client information system and forwarded to patient information system to enable all who are providing care to the patient to share knowledge.

How ready the current organisation is to undertake a change of this kind requires an evaluation of these changes: which would be regarded as positive (by whom) and which would be regarded as problematic (by whom) and might become barriers to the development?


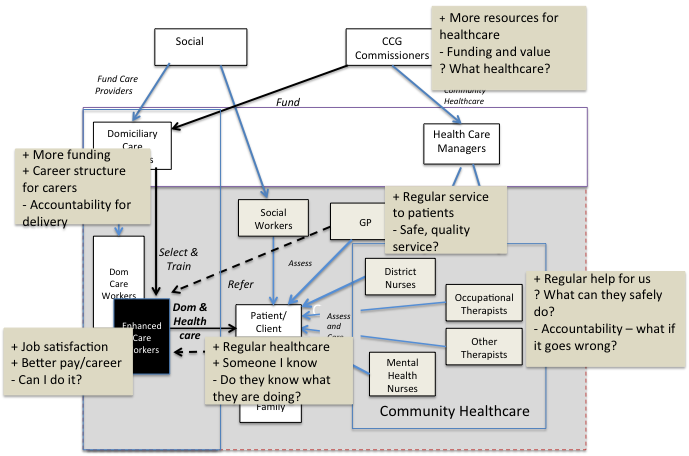


*Figure 12*

*Illustrative Evaluations of the ‘To Be’ System by Major Role Holders*

Figure 12 provides an illustrative account of how these changes might be viewed, both as positive and negative developments, by the main role holders in the target system and in adjoining systems. Ideally, these assessments would be made by the role-holders themselves or people close to them. Because of the pandemic there was no opportunity to engage with the staff concerned but the initial work in the two boroughs provided information upon which to make these assessments.

As a result of this process, the list of elements in the local work system (e.g. the work roles, processes and technical systems) could be entered into the first part of the checklist (figure 6), changes identified and evaluations as in figure 12 entered.

As another product of the project we have produced a version of the ‘readiness for change’ assessment process which can be used by people responsible for local system developments to assess the impact an innovation might have on the work system (Eason and Maton-Howarth 2020).

**7. Conclusions**

The initial studies in WORKTECC identified many recent change programmes and enabled a checklist to be constructed that covered the factors likely to influence readiness to change in community healthcare services. The checklist covers both Distal factors, those that relate to the whole work system, and Proximal factors, those that relate to the local system that will be impacted by a specific innovation. In order to establish the factors specific to the local system another analysis stage has been introduced that overlays a description of the current work system with an account of what changes would be required if the innovation was made. The checklist has now been used to assess the readiness for change in one of the boroughs for the OR workforce innovations identified in the project.

**References**

Carayon  P, Wooldridge  A, Hoonakker  P, et al (2020) ‘SEIPS 3.0: Human-centered design of the patient

Journey for patient safety’ *Applied Ergonomics* 2020;84:103033 [doi:10.1016/j.apergo.2019.103033](http://dx.doi.org/10.1016/j.apergo.2019.103033)pmid:http://www.ncbi.nlm.nih.gov/pubmed/31987516

Checkland P. (1981). *Systems Thinking, Systems Practice*. Wiley.

Eason K.D. Harker S.D.P. & Olphert C.W. (1996) ‘Representing Socio-Technical Systems Options in the Development of New Forms of Work Organization’ *European Journal of Work and Organizational Psychology* 5:3 399-420

Eason K.D. (2007) ‘Local sociotechnical system development in the NHS National Programme for Information Technology’ *Journal of Information Technology* 22 (3) 257-264

Eason K. D. (2014) ‘Afterword: The past, present and future of sociotechnical systems theory’ *Applied Ergonomics* 45(2A) 213-220 DOI: 10.1016/j.apergo.2013.09.017

Eason K.D. & Maton-Howarth W. (2020) ‘The Planning for Change Framework’ https://www.bayswaterinst.org/2020/10/30/the-law-of-unexpected-consequencesdownload-our-guide-to-spotting-knock-on-effects-in-advance/

# Emery F. E. and Trist E.L. (1965) ‘The Causal Texture of Organizational Environments’ *Human Relations*, Feb 1 [https://doi.org/10.1177/001872676501800103](https://doi.org/10.1177%2F001872676501800103)

Herbst P. G. (1974) *Socio-technical design* London, Tavistock Publications

Holt, D.T., Armenakis, A.A., Feild, H.S., Harris, S.G. (2007). ‘Readiness for organizational change: the systematic development of a scale’. *Journal of Applied Behavioral Science*, 43(2), 232-255.

Katz D and Kahn R L.(1966) ‘*The social psychology of organizations.’*
New York: Wiley, 1966.

Nohria N and Gulati R (1997) ‘What is the optimum amount of organizational slack?: A study of the relationship between slack and innovation in multinational firms’ *European Management Journal* 15:6  December 1997 603-611

Roberts C. & Geels  F.W. (2019) ‘Conditions and intervention strategies for the deliberate acceleration of socio-technical transitions: lessons from a comparative multi-level analysis of two historical case studies in Dutch and Danish heating,’ *Technology Analysis & Strategic Management*, 31:9, 1081-1103, DOI: 10.1080/09537325.2019.1584286

Senge, Peter M. (1990), [*The Fifth Discipline*](https://books.google.com/books?id=bVZqAAAAMAAJ), Doubleday/Currency, [ISBN](https://en.wikipedia.org/wiki/ISBN_(identifier)) [0-385-26094-6](https://en.wikipedia.org/wiki/Special:BookSources/0-385-26094-6)
