# Supplementary material for: A mixed methods approach for the identification and assessment of workforce innovations in home health care
Source: Front Health Serv. 2026 Jul 16;6:1749947. doi: 10.3389/frhs.2026.1749947 (PMC13422396; doi:10.3389/frhs.2026.1749947)
Supplement: Supplementary File S8 — Summary of sociotechnical systems analysis results. [file Datasheet8.docx]

**Sociotechnical systems analysis summary conclusions**

| **Innovation** | **Enablers** | **Inhibitors** | **Neutral observations** |
| --- | --- | --- | --- |
| 1. Specialisation | - Aligned with current professional development practices - Senior staff may welcome focus on most complex cases - Requires little technical development or additional data | - Counter to valued continuity of care in mental health - Staff shortages and absence may undermine fidelity - More staff per patient jeopardises liaison with social care - May have consequences for team composition and training | More visits for some patients |
| 2a. Generalisation - within discipline |  | - Senior staff may view as involving mundane work - Senior staff may view as counter to career progression - Requires support of professional associations - Staff shortages and absence may undermine fidelity - May have consequences for team composition - May require refresher training and retraining | Could implement 2a and gradually broaden duties towards greater generalisation (2b), e.g. first reduce duplication in patient assessments |
| 2b. Generalisation - across disciplines | - Aligned with local and national aim for less fragmented care | - Counter to many current organisational and cultural norms - Staff may view as counter to career progression - Physical care counter to professional identity in mental health nursing - Efforts to introduce more general roles have struggled elsewhere - Refresher training may constitute appreciable workload |  |
| 3. Balancing workload through districting | - Could lead to improvements in staff wellbeing - The calculation of new districts would not need staff engagement - Future use could account for anticipated demographic change | - Little appetite as districts agreed in 2018 across multiple organisations - Re-deployment of staff to new districts subject to extensive consultation - Could fracture working relationships with GPs, social workers etc. - Could disrupt continuity of care for some patients - Potential instability of service over period of change |  |
| 4. Balancing workload through team size and composition | - Fairer workload balance may improve staff wellbeing | - Scope for redeployment or redundancy processes impacting morale - Might be challenging/expensive to fill some roles - Might disrupt working relationships with other community services - Could disrupt continuity of care for some patients - Might require periodic adjustment as demand changes |  |
| 5. Balancing workload through allocation with fuzzy boundaries | - Fairer workload balance may improve staff wellbeing - No major re-organisation needed - Can accommodate gradual shifts in patterns of demand | - Requires effective liaison and information sharing between teams - Depends on daily use of technological solution to revise allocations - ‘Change fatigue’ may result in passive, minimal acceptance - Staff may resent covering for teams seen as ‘underperforming’ - Could disrupt continuity of care for some patients |  |
| 6. Enhancing continuity of care through automated allocation | - Could reduce senior time spent on allocation process - Would support staff having a longer "planning horizon" - Would standardise approach across teams / allocators - Would not require a major change programme - More continuity of care aligned with patient preferences - More continuity of care may improve outcomes - Continuity of care valued by staff and prioritised for some patients - Allocators may welcome being free of burdensome task | - Relies on accurate information about roster and staff competencies - Some local facets of problem may not be captured within algorithm - Late changes to demand/staffing may entail manual adjustment - May not be adopted if substantial manual adjustment required - Investment required for software and data pipeline - May be resisted if solutions too divergent from current practice - Allocators may lose oversight of the caseload and staff workloads - District nurses may resent loss of autonomy for own schedule - Allocators may resent loss of status / power that comes with role - Staff shortages and reliance on agency staff may undermine ambition |  |
| 7. Minimising cost through automated allocation | - Could reduce senior time spent on allocation process - Would support staff having a longer "planning horizon" - Would standardise approach across teams / allocators - Would not require a major change programme - Responsive to budgetary pressures - Allocators may welcome being free of burdensome task | - Relies on accurate information about roster and staff competencies - Some local facets of problem may not be captured within algorithm - Late changes to demand/staffing may entail manual adjustment - May not be adopted if substantial manual adjustment required - Investment required for software and data pipeline - Allocators may resent loss of status / power that comes with role - May be resisted if solutions too divergent from current practice - District nurses may resent loss of autonomy for own schedule - Allocators may lose oversight of the caseload and staff workloads |  |
| 8. Balancing continuity of care and costs through automated allocation | - Could reduce senior time spent on allocation process - Would support staff having a longer "planning horizon" - Would standardise approach across teams / allocators - Would not require a major change programme - More continuity of care aligned with patient preferences - More continuity of care may improve outcomes - Continuity of care valued by staff and prioritised for some patients - Responsive to budgetary pressures - Allocators may welcome being free of burdensome task | - Relies on accurate information about roster and staff competencies - Some local facets of problem may not be captured within algorithm - Late changes to demand/staffing may entail manual adjustment - May not be adopted if substantial manual adjustment required - Investment required for software and data pipeline - May be resisted if solutions too divergent from current practice - Allocators may lose oversight of the caseload and staff workloads - District nurses may resent loss of autonomy for own schedule - Allocators may resent loss of status / power that comes with role - Staff shortages / reliance on agency staff may undermine continuity |  |
