# Supplementary material for: A mixed methods approach for the identification and assessment of workforce innovations in home health care
Source: Front Health Serv. 2026 Jul 16;6:1749947. doi: 10.3389/frhs.2026.1749947 (PMC13422396; doi:10.3389/frhs.2026.1749947)
Supplement: Supplementary File S2 — Rich Picture. [file Datasheet2.pdf]

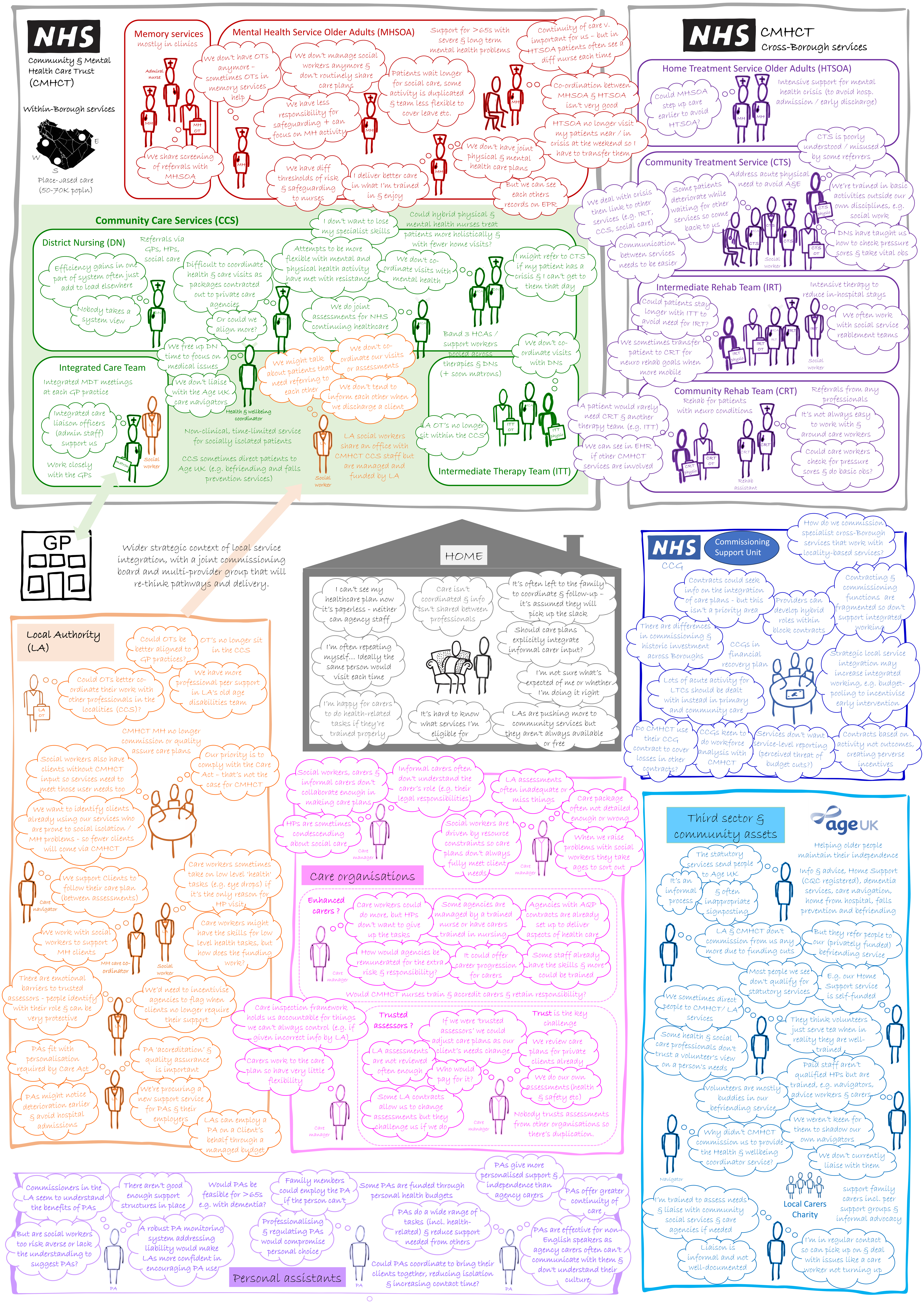
NHS
Community & Mental Health Care Trust (CMHCT)
Within-Borough services
E
W
S
Place-based care (50-70K popln)
Memory services
mostly in clinics
We don't have OTs anymore - sometimes OTs in memory services help
We share screening of referrals with MHSOA
Admiral nurse
MH
MH OT
Mental Health Service Older Adults (MHSOA)
Support for >65s with severe & long term mental health problems
We don't manage social workers anymore & don't routinely share care plans
Patients wait longer for social care, some activity is duplicated & team less flexible to cover leave etc.
We have less responsibility for safeguarding + can focus on MH activity
We have diff thresholds of risk & safeguarding to nurses
I deliver better care in what I'm trained in & enjoy
Continuity of care v. important for us - but in HTSOA patients often see a diff nurse each time
Co-ordination between MHSOA & HTSOA isn't very good
HTSOA no longer visit my patients near / in crisis at the weekend so I have to transfer them
We don't have joint physical & mental health care plans
But we can see each others records on EPR
NHS
CMHCT
Cross-Borough services
Home Treatment Service Older Adults (HTSOA)
Could MHSOA step up care earlier to avoid HTSOA?
Intensive support for mental health crisis (to avoid hosp. admission / early discharge)
CTS is poorly understood / misused by some referrers
Community Treatment Service (CTS)
Address acute physical need to avoid A&E
We deal with crisis then link to other services (e.g. IRT, CCS, social care)
Some patients deteriorate while waiting for other services so come back to us
We're trained in basic activities outside our own disciplines, e.g. social work
DNs have taught us how to check pressure sores & take vital obs
Communication between services needs to be easier
CTS
CTS physio
Social worker
Intermediate Rehab Team (IRT)
Intensive therapy to reduce in-hospital stays
Could patients stay longer with ITT to avoid need for IRT?
We often work with social service reablement teams
We sometimes transfer patient to CRT for neuro rehab goals when more mobile
IRT
IRT OT
IRT physio
Social worker
Community Rehab Team (CRT)
Referrals from any professionals
Rehab for patients with neuro conditions
A patient would rarely need CRT & another therapy team (e.g. ITT)
It's not always easy to work with & around care workers
We can see in EHR if other CMHCT services are involved
Could care workers check for pressure sores & do basic obs?
CRT
CRT OT
CRT physio
Rehab assistant
Community Care Services (CCS)
I don't want to lose my specialist skills
Could hybrid physical & mental health nurses treat patients more holistically & with fewer home visits?
District Nursing (DN)
Referrals via GPs, HPs, social care
Efficiency gains in one part of system often just add to load elsewhere
Nobody takes a system view
Difficult to coordinate health & care visits as packages contracted out to private care agencies
Or could we align more?
Attempts to be more flexible with mental and physical health activity have met with resistance
We don't co-ordinate visits with mental health
We do joint assessments for NHS continuing healthcare
I might refer to CTS if my patient has a crisis & I can't get to them that day
DN
HCA
We free up DN time to focus on medical issues
Integrated Care Team
Integrated MDT meetings at each GP practice
Integrated care liaison officers (admin staff) support us
Work closely with the GPs
Matron
Social worker
We don't liaise with the Age UK care navigators
Health & wellbeing coordinator
We might talk about patients that need referring to each other
We don't co-ordinate our visits or assessments
We don't tend to inform each other when we discharge a client
Band 3 HCAs / support workers pooled across therapies & DNs (+ soon matrons)
We don't co-ordinate visits with DNs
A OT's no longer sit within the CCS
ITT OT
ITT physio
Intermediate Therapy Team (ITT)
Non-clinical, time-limited service for socially isolated patients
CCS sometimes direct patients to Age UK (e.g. befriending and falls prevention services)
LA social workers share an office with CMHCT CCS staff but are managed and funded by LA
Social worker
GP
Wider strategic context of local service integration, with a joint commissioning board and multi-provider group that will re-think pathways and delivery.
HOME
I can't see my healthcare plan now it's paperless - neither can agency staff
Care isn't coordinated & info isn't shared between professionals
It's often left to the family to coordinate & follow-up - it's assumed they will pick up the slack
Should care plans explicitly integrate informal carer input?
I'm often repeating myself... Ideally the same person would visit each time
I'm not sure what's expected of me or whether I'm doing it right
I'm happy for carers to do health-related tasks if they're trained properly
It's hard to know what services I'm eligible for
LAs are pushing more to community services but they aren't always available or free
NHS
Commissioning Support Unit
CCG
How do we commission specialist cross-Borough services that work with locality-based services?
Contracts could seek info on the integration of care plans - but this isn't a priority area
Providers can develop hybrid roles within block contracts
Contracting & commissioning functions are fragmented so don't support integrated working
There are differences in commissioning & historic investment across Boroughs
CCGs in financial recovery plan
Strategic local service integration may increase integrated working, e.g. budget-pooling to incentivise early intervention
Lots of acute activity for LTCs should be dealt with instead in primary and community care
Do CMHCT use their CCG contract to cover losses in other contracts?
CCGs keen to do workforce analysis with CMHCT
Services don't want service-level reporting (perceived threat of budget cuts?)
Contracts based on activity not outcomes, creating perverse incentives
Local Authority (LA)
Could OTs be better aligned to GP practices?
OT's no longer sit in the CCS
Could OTs better co-ordinate their work with other professionals in the localities (CCS)?
We have more professional peer support in LA's old age disabilities team
LA OT
CMHCT MH no longer commission or quality assure care plans
Social workers also have clients without CMHCT input so services need to meet those user needs too
Our priority is to comply with the Care Act - that's not the case for CMHCT
We want to identify clients already using our services who are prone to social isolation / MH problems - so fewer clients will come via CMHCT
We support clients to follow their care plan (between assessments)
Care navigator
Care workers sometimes take on low level 'health' tasks (e.g. eye drops) if it's the only reason for HP visit
We work with social workers to support MH clients
MH care co-ordinator
Social worker
Care workers might have the skills for low level health tasks, but how does the funding work?
There are emotional barriers to trusted assessors - people identify with their role & can be very protective
We'd need to incentivise agencies to flag when clients no longer require their support
PAs fit with personalisation required by Care Act
PA 'accreditation' & quality assurance is important
We're procuring a new support service for PAs & their employers
PAs might notice deterioration earlier & avoid hospital admissions
LAs can employ a PA on a client's behalf through a managed budget
Social workers, carers & informal carers don't collaborate enough in making care plans
Informal carers often don't understand the carer's role (e.g. their legal responsibilities)
LA assessments often inadequate or miss things
HPs are sometimes condescending about social care
Care manager
Social workers are driven by resource constraints so care plans don't always fully meet client needs
Care package often not detailed enough or wrong
When we raise problems with social workers they take ages to sort out
Care manager
Care organisations
Enhanced carers?
Care workers could do more, but HPs don't want to give up the tasks
Some agencies are managed by a trained nurse or have carers trained in nursing
Agencies with A&P contracts are already set up to deliver aspects of health care
How would agencies be remunerated for the extra risk & responsibility?
It could offer career progression for carers
Some staff already have the skills & more could be trained
Care manager
Would CMHCT nurses train & accredit carers & retain responsibility?
Care inspection framework holds us accountable for things we can't always control (e.g. if given incorrect info by LA)
Carers work to the care plan so have very little flexibility
Trusted assessors?
If we were 'trusted assessors' we could adjust care plans as our client's needs change
Trust is the key challenge
We review care plans for private clients already
LA assessments are not reviewed often enough
Who would pay for it?
We do our own assessments (health & safety etc)
Some LA contracts allow us to change assessments but they challenge us if we do
Care manager
Care manager
Nobody trusts assessments from other organisations so there's duplication.
Third sector & community assets
ageUK
Helping older people maintain their independence
The statutory services send people to Age UK
It's an informal process
& often inappropriate signposting
Info & advice, Home Support (CQC registered), dementia services, care navigation, home from hospital, falls prevention and befriending
LA & CMHCT don't commission from us any more due to funding cuts
But they refer people to our (privately funded) befriending service
Most people we see don't qualify for statutory services
E.g. our Home Support service is self-funded
We sometimes direct people to CMHCT / LA services
Some health & social care professionals don't trust a volunteer's view on a person's needs
They think volunteers just serve tea when in reality they are well-trained
Paid staff aren't qualified HPs but are trained, e.g. navigators, advice workers & carers
Volunteers are mostly buddies in our befriending service
We weren't keen for them to shadow our own navigators
Why didn't CMHCT commission us to provide the Health & wellbeing coordinator service?
We don't currently liaise with them
Navigator
Local Carers Charity
Support family carers incl. peer support groups & informal advocacy
I'm trained to assess needs & liaise with community social services & care agencies if needed
Liaison is informal and not well-documented
I'm in regular contact so can pick up on & deal with issues like a care worker not turning up
Commissioners in the LA seem to understand the benefits of PAs
There aren't good enough support structures in place
Would PAs be feasible for >65s e.g. with dementia?
Family members could employ the PA if the person can't
Some PAs are funded through personal health budgets
PAs give more personalised support & independence than agency carers
PAs offer greater continuity of care
But are social workers too risk averse or lack the understanding to suggest PAs?
A robust PA monitoring system addressing liability would make LAs more confident in encouraging PA use
Professionalising & regulating PAs would compromise personal choice
PAs do a wide range of tasks (incl. health-related) & reduce support needed from others
PAs are effective for non-English speakers as agency carers often can't communicate with them & don't understand their culture
Could PAs coordinate to bring their clients together, reducing isolation & increasing contact time?
Personal assistants
PA
PA
PA
